# Leasing or Selling? Durable Goods Manufacturer Marketing Model Selection under a Mixed Carbon Trading-and-Tax Policy Scenario

**DOI:** 10.3390/ijerph16020251

**Published:** 2019-01-16

**Authors:** Yuxiang Zhang, Deqing Tan, Zhi Liu

**Affiliations:** 1 School of Economics and Management, Southwest Jiaotong University, Chengdu 610031, China; 2 College of Management Engineering, Anhui Polytechnic University, Wuhu 241000, China; liuzhi@nuaa.edu.cn

**Keywords:** mixed carbon policy, monopoly manufacturer, durable goods, consumption behavior, leasing versus selling, win-win result

## Abstract

Many carbon reduction policies have been implemented to reduce carbon dioxide in the manufacturing process of products. However, many products emit more carbon dioxide in the consumption process. From the consumer’s utility perspective, this paper firstly analyses the manufacturing and marketing model selection decisions of a monopoly manufacturer under the mixed carbon policy, and then a win-win result that can encourage the manufacturer to choose the marketing model with lower carbon emissions while at the same time obtaining the optimal profit is discussed. The results show that the production activity will proceed only when the carbon trading price is lower than a certain threshold. When the carbon trading price is lower than a certain threshold, leasing represents the manufacturer’s optimal marketing model. When the carbon trading price is higher than the threshold, selling represents the manufacturer’s optimal marketing model. For the carbon cap *Q*, there are equilibrium intervals in which the government can achieve the aim of controlling carbon emissions, while not overly affecting the manufacturer’s enthusiasm for production. For the carbon trading price and the carbon tax rate, there are two different intervals in which leasing gains more profit for the manufacturer while emitting lower carbon emissions.

## 1. Introduction

After the climate conference in Copenhagen, carbon reduction policy has been considered a significant mechanism to reduce the greenhouse effect all over the world [1]. There are many widely used carbon policies, such as carbon cap-and-trade systems, carbon emissions taxes and carbon emissions offset policies [2]. Before 2008, many developed countries, such as the United Kingdom, Germany, Norway, and Finland, had adopted a carbon tax as their low-carbon policy. In 2008, the European Union formally launched the Emission Trade System, which currently is the largest carbon trading market. Then, a carbon cap-and-trade policy was implemented in many countries, such as the United States (some individual states) and China [3,4,5]. The aim of all these carbon policies was to limit the carbon emissions in the production process.

However, too much attention has been paid to the implementation of low-carbon policies that reduce the impact of production on the environment. Few carbon policies have focused on the production and consumption process at the same time. Approximately 30%–40% of the decline in ecological quality created by global warming is due to the household durable goods consumption [6]. In real life, many durable goods, such as automobiles, air conditioners, and refrigerators, emit more carbon dioxide during the consumption process [7]. For example, for the electric automobile, which is currently heavily promoted, the carbon emissions per unit in the production process are approximately 70 g/km, and the carbon emissions per unit in the consumption process are approximately 188 g/km [8]. This means that even for an electric vehicle, carbon emissions in the consumption process occupied more than 70% of its total carbon emissions in the lifecycle. With the further promotion of future carbon emissions reduction policies, the durable products with high carbon emissions in both production and consumption processes will be restricted by not just one single carbon policy. A mixed carbon policy that limits carbon emissions in both the production and the consumption process should be implemented in the future.

The implementation of a carbon policy has brought great challenges to the high carbon emissions durable goods manufacturer. In the classical durable goods theory, a leasing strategy is always the optimal market strategy for the monopoly manufacturer [9]. Many scholars have extended this classical durable goods theory finding that many external factors affect the manufacturer’s choice between leasing and selling [10,11,12]. With the implementation of the mixed carbon policy, whether leasing is still the optimal marketing strategy of the monopoly manufacturer is a question worth investigating. A durable goods manufacturer had to regard carbon policy as an important influencing factor in the selection strategy between leasing and selling.

Meanwhile, the leasing strategy is widely regarded as a greener marketing model rather than one based on a selling strategy [13,14]. Many enterprises, such as Hewlett-Packard (HP), Bavarian Motor Works (BMW), and GREE Electric Appliances (GREE), have improved their green image through the introduction of leasing programmes. However, whether this conclusion is still valid under the implementation of carbon policy remains to be verified.

Therefore, the authors investigate with respect to a mixed carbon policy, the manufacturer’s selection of an optimal marketing model strategy when choosing between leasing and selling. Furthermore, comparing leasing and selling, this paper determines which strategy is greener. Specifically, the following questions are addressed in this paper:

Main question: Do specific intervals exist for the carbon trading price and carbon tax rate that can achieve a win-win result between the monopoly manufacturer’s profit and a friendly environment?

Other related questions: (1) How do the manufacturer’s production and the consumers’ consumption activities change after the implementation of the mixed carbon policy? (2) What will happen to the optimal leasing and selling profit of the manufacturer under the mixed carbon policy? How do the carbon trading price, the carbon cap, and the carbon tax rate affect the leasing and selling profit? (3) Under a mixed carbon policy, what is the manufacturer’s optimal marketing model selection strategy?

The main contribution of this paper is that it proposes a mixed carbon policy that targets the durable goods’ carbon emissions in both the production and the consumption process. Additionally, this paper provides new analysis results regarding how a manufacturer adjusts its optimal marketing model between leasing and selling. Moreover, by comparing the manufacturer’s profits and total carbon emission volume, this paper also offers some effective policy suggestions that guide manufacturers to choose the marketing model with lower carbon emissions.

To answer the questions above, this paper aims to examine the monopoly manufacturer’s optimum production quantity decisions made under the constraints of a low carbon policy for both production and consumption processes and analyses their effects on the leasing and selling profit. Additionally, this paper, based on the condition that the manufacturer obtains the optimal profits, explores the carbon trading price and the carbon tax rate intervals that enable the manufacturer to choose the more profitable marketing strategy and at the same time to achieve a reduction in carbon emissions to the environment.

The reminder of this paper is structured as follows: The relevant literature is reviewed in Section 2. In Section 3, the parameters and decision variables are described and the basic models proposed in this paper are then presented. In Section 4, the model formulation is described and the optimal solutions is revealed under leasing and selling. In Section 5, the changing characteristics of consumption behaviour under the mixed carbon policy are discussed. The optimum quantity and marketing model selection strategy for the monopolistic manufacturer are discussed; then, by comparing the actual carbon emissions of leasing and selling, a strategy for obtaining a win-win result is presented. In Section 6, the numerical analysis and results are described. In Section 7, the whole paper is summarized, and the implications of this research are discussed.

## 2. Literature Review

This paper proposes a mixed carbon policy that restrains the durable goods’ carbon emissions in both the production and the consumption process. In addition, the authors aim to investigate the impact of the mixed carbon policy on the consuming behaviour with respect to the durable goods and on the manufacturer’s selection of an optimal marketing model selection strategy when choosing between leasing and selling. As such, three streams of studies relevant to our work are reviewed as follows: the carbon cap-and-trade versus the carbon tax policy, a leasing versus a selling strategy of the monopoly manufacturer, and the impacts of carbon policies on the manufacturer’s production and operation management.

### 2.1. Carbon Cap-and-Trade versus Carbon Tax

Academic research has recently focused on the impact of different carbon policies, such as carbon cap-and-trade [5,15,16,17,18,19,20], and carbon taxes [21,22,23,24,25,26,27,28]. All of these existing studies investigate the change of the manufacturer’s decisions before and after the introduction of a single carbon policy. Carbon cap-and-trade is a market-based carbon policy for controlling carbon emissions [4]. In a carbon cap-and-trade system, a manufacturer is initially allocated a quota for a fixed number of carbon emissions over a single period. If the carbon emission volume is below the emissions permit, the manufacturer can sell its unused carbon emissions quota in the carbon trade market. Moreover, if the carbon emission volume is higher than their emissions permit, to avoid a heavy penalty, the manufacturer has to buy from the carbon trade market the corresponding permits to fill the gap. The carbon tax is an incentive-based carbon policy that levied a fixed tax rate on carbon dioxide emissions [4,29].

Furthermore, many scholars have been intensely interested in debating the merits of the carbon cap-and-trade and the carbon tax policies [30,31,32,33]. Avi-Yonah et al. [34] believed that carbon tax is easier to implement than carbon cap-and-trade, and easier to adjust according to the actual situation of the market. Moreover, the implementation cost of carbon tax policy is very low. Keohane et al. [35] summarized three advantages of carbon cap-and-trade over carbon tax: efficiency advantage, political advantage, flexible advantage.

Wei et al. [33] used bibliometric method to compare the merits of four carbon policies: command-and control, quantity-based, price-based, and hybrid. He et al. [4] constructed a generation expansion planning (GEP) model to compare the effectiveness and efficiency between carbon cap-and-trade policy and carbon tax policy. And the numerical analysis results showed that both carbon policies have their own advantages, there is not a clear winner. Li et al. [32] used computable general equilibrium (CGE) model to analyze the impact of carbon tax, carbon cap-and-trade and mixed carbon policy on China’s carbon emissions based on 17 scenarios in China. The results showed that single carbon cap-and-trade or carbon tax cannot achieve China’s carbon emission reduction commitment in 2030, thereby the mixed carbon policy become the inevitable choice. Moreover, under the mixed carbon policy, low carbon tax rate is not recommendation.

### 2.2. Leasing versus Selling Selection of Durable Goods

Leasing versus selling is an interesting question from a carbon reduction perspective. The study of durable goods leasing versus selling began with the Coase paradox [9], which is one of the classic topics of durable goods. Based on his research, many scholars at home and abroad have carried on extensive studies on the profitability of the leasing versus selling decision for durable goods and have obtained rich theoretical results [36,37,38]. Desai et al. [37] studied the impact of devaluation on a durable goods leasing and selling strategy and found that when the depreciation rate of durable goods exceeds a certain value, the monopolist will switch to a selling strategy. Moreover, Desai et al. [10] compared the profitability of leasing and selling under competitive market conditions. The results showed that the manufacturer would choose a leasing-selling mixed strategy under competitive market conditions and that with the increases of competition intensity, the manufacturer eventually adopts a pure selling strategy. Sreekumar et al. [38] analysed how independent producers of complementary products affect the leasing and selling strategies of durable goods manufacturers. Their study found that durable goods manufacturers abandoned the pure leasing strategy and in turn chose a leasing-selling mixed strategy or pure selling strategy instead, due to the presence of complementary products by independent producers. Then, Sreekumar et al. [12] further deepening the examination of marketing structure, explored its impacts on the leasing and selling profit of several competing distributors. Chien et al. [11] explored the impact of network effects on the leasing and selling profit of monopoly manufacturers. Their research found that under the influence of a network effect, the monopoly manufacturer could gain more profits from the selling strategy rather than from the leasing strategy.

In comparing the two strategies, determining whether leasing or selling is the more environmentally superior strategy is an interesting topic, and the conclusion is still unclear. Many scholars hold the opinion that leasing rather than selling is more environment-friendly [13,39]. Because under a leasing strategy, the monopoly manufacturer maintains ownership of the durable goods. In such a case, the monopoly manufacturer has the incentive to remarket the used durable good, thereby hindering the production of new products and reducing the environmental impact related to manufacturing new products. However, from the perspective of durability, based on the fact that the average use durations of used durable goods under leasing are clearly shorter than those under a selling strategy, some scholars believe that leasing is not more environmentally friendly than selling [40,41]. Leasing will cause the monopoly manufacturer to remarket the leased used product earlier than they would under a selling strategy. Therefore, leasing ends up being more environmentally unfriendly than selling. Agrawal et al. [42] based on the profit maximization of the monopoly manufacturer, explored whether leasing is a more profitable and environmentally friendly marketing strategy. The results showed that whether leasing is greener than selling depends on the removal timing. Then, Agrawal et al. [7] considering the monopoly manufacturer that offers a trade-in programme, investigated the same question.

### 2.3. Production and Operation Management under Carbon Policies

In recent years, with the enhancement of the consumers’ awareness of environmental protection and the implementation of energy conservation and emissions reduction policies, academics have carried on an extensive and fervent discussion of the enterprise’s decision-making process under an environmental restriction [5,43,44,45,46]. Therefore, many scholars at home and abroad began to pay attention to the impact of low carbon environmental protection on a durable goods strategy. Chen et al. [47] analysed the interaction of low carbon preference, consumer products strategy and government environmental standards and gained two novel conclusions: low carbon product development and stringent environmental standards would not be necessarily beneficial to the environment. Dobos et al. [48] studied the optimal production-inventory strategies for a company under the cap-and-trade policy regulation and found that the optimal production quantities are reduced after applying the emissions trading policy. Benjaafar et al. [49] analysed the optimal production decisions covering multiple periods under a carbon tax, a cap-and-trade policy, and under a carbon offsets policy: the results showed that the cap allocated by the government has no effect on the firm’s optimal decisions. Cohen et al. [50] analysed the impact of government subsidies for low-carbon technology on the production and pricing of durable goods manufacturers. Xu et al. [51] studied the production and carbon emissions reduction level in a make-to-order supply chain under cap-and-trade regulation and found that both the wholesale price and cost-sharing contracts can coordinate the supply chain.

From the above literature, we found that under a carbon cap-and-trade policy, manufacturers can adjust their production strategy flexibly by selling or buying carbon quotas, but the total volume of carbon emissions is limited by a pre-set carbon cap. Furthermore, due to the tradability of carbon quotas, companies are more motivated to reduce carbon emissions under the carbon cap-and-trade policy. In contrast, under a carbon tax, manufacturers impose the tax for each unit of the product that emits carbon dioxide. The carbon tax is an easier and lower cost policy than the carbon trade-and-cap policy. Both policies are currently implemented separately in different countries; however, there is no effective restriction on products with high carbon emissions in both the production and the consumption process. Thus, the current carbon policies are no longer able to provide effective production and marketing advice to the manufacturer. It is necessary and valuable to study how a manufacturer should adjust its strategy under a mixed carbon policy that curbs carbon emissions in both the production and the consumption process. Moreover, the above research studies are mainly focused on the production inventory decision and the emission reduction strategies of the supply chain enterprises under a low carbon strategy. These studies only considered how to achieve maximum profits from the manufacturer’s perspective, and no consideration was given as to whether it would truly reduce the carbon emissions.

To address these issues, considering the emission of carbon dioxide in both the production and the consumption process of durable goods, this paper builds a durable goods monopoly manufacturer’s leasing and selling model. Initially, this paper introduces a mixed carbon policy, which includes a carbon trade-and-cap policy (constraint manufacturer) and a carbon tax policy (constraint customers), and investigates the effects of the mixed carbon policy on the manufacturer’s production, profits and marketing model selection. In addition, different from the previous literature, this paper tries to find a win-win result between the achieving of the manufacturer’s profit and the maintaining of a friendly environment.

## 3. Problem Description and Notations

### 3.1. Problem Description

We develop a discrete-time, infinite-horizon decision-making model in which a profit-maximizing monopoly manufacturer produces and offers a single durable goods to consumers. Additionally, the government uses a mixed carbon policy to restrain the manufacturer’s production and marketing decisions.

We assume that the durable goods can offer two periods of service to consumers. The product deteriorates with use and has a finite durability [7,10,42]. We refer to a product in its first period of useful life as new (denoted by the subscript *n*) and refer to the quality of the new durable goods as *v*. We refer to the product’s second period of useful life as used (denoted by the subscript u) and refer to the quality of the used durable goods as *δ_v_*, *δ* ∈ (0, 1). We refer to *δ* as the product’s durability [7,52]. After two periods of service, the product is fully deteriorated.

The monopoly manufacturer has two marketing model options: either lease or sell its products to consumers. The characters *l* and *s* denote parameters specific to the leasing and selling, respectively. Under the leasing model, the monopoly manufacturer offers one period operating leases in which the monopolist maintains ownership of the durable products. The rental price of the new product and used product is *p_ln_* and *p_lu_*, respectively. After one period of service, the new product becomes used, and it will be rented out as a used product for the next period. Additionally, the used product eventually became useless, not available for further renting. Under the selling model, the monopolist only sells new products in which the monopolist no longer maintains ownership of the durable products. The market selling price is *p_sn_*; used products are traded between consumers on the secondary market at the market clearing price *p_su_*.

The product emits carbon dioxide in both the production and the consumption process. *e*_0_ denotes the carbon dioxide emissions of producing one durable goods. In addition, the carbon dioxide emissions of consuming one new and one used durable goods is denoted by *e*_1_ and *e*_2_, respectively. We allow *e_p_* < *e*_1_ ≤ *e*_2_ [44]. It is commonly observed in practical life that the carbon dioxide emissions in the consumption process are far higher than the carbon dioxide emissions in the production process are. Additionally, similar to cars and refrigerators, as the product depreciates, its carbon dioxide emissions increase. The cost of emitting carbon dioxide emissions is borne by the one who owns the products.

We consider two processes for carbon dioxide emissions: production and consumption. Carbon dioxide emissions in both processes are constrained by carbon policies. Under the condition stipulated in the carbon cap-and-trade policy, the government allocates an emission quota *Q* to the manufacturer. If the actual carbon dioxide emissions are larger (less) than *Q*, then the manufacturer needs(can) to buy(sell) carbon dioxide emission credits from (into) the carbon trading market with a unit carbon trading price $p_{e}$. Furthermore, we assume that the government will introduce a carbon tax policy to reduce the carbon emissions emitted by the products of which consumers remain ownership. *λ* (*λ* > 0) denotes the carbon tax of per unit carbon emitted during the consumption.

According to previous research, a carbon cap-and-trade policy is more effective than a carbon tax policy in reducing carbon emissions and stimulating the enthusiasm of manufacturers to reduce carbon emissions [32,35]. In this case, under the leasing strategy, as consumers do not own the durable goods, all the expenses and taxes on carbon emissions both in the production and in the consumption process are borne by the monopoly manufacturer. Therefore, through the perspective of social welfare, we assume that carbon emissions in both the production and the consumption process are constrained by the carbon cap-and-trade policy. Under the selling strategy, the manufacturer covers the expenses of carbon emissions in the production process. The tax on carbon emissions during consumption is borne by the consumers because they own the durable goods.

The size of the consumer population remains constant over time and is normalized to 1. Consumers are heterogeneous in the utility they derive from consumption and are characterized by their type *θ*, which is time-independent and finite. *θ* is uniformly distributed over [0,1] [37,42]. The consumer θ’s utility derived from one-period of use of the new product, one-period of use of the used product, and of remaining inactive for one period is given by $u_{n}\left(\theta \right)$, $u_{u}\left(\theta \right)$, and 0, respectively. Furthermore, we assume that one consumer only rents or buys one durable good at a time. Consumers are dependent on the service provided by the monopoly manufacturer, and they will rent or buy the durable goods repeatedly once they use them.

Other assumptions are made as follows: *ρ* represents the discount factor of revenues or cash flows received in the next period, *ρ* ∈ (0, 1); the periods are indexed by *t* ≥ 0; to simplify the analysis, we assume the marginal production cost of the monopoly manufacturer is 0; the transaction costs in either market is 0.

### 3.2. Notations

Table 1 summarizes the main notations used in this paper. The superscript “*” represents optimal values of the variables. In addition, the superscript “’” represents values without the mixed carbon policy.

## 4. Model Formulation and Solution

### 4.1. Leasing Model

Under the leasing model, the monopolist offers single period leasing services of new and used durable goods. Therefore, we can partition the consumers into three different groups: on the interval $\left[\theta_{l1},1\right]$, the consumers who only rent new durable goods in every period; on the interval $\left[\theta_{l2},\theta_{l1}\right)$, the consumers who only rent used durable goods in every period; and on the interval $\left[0,\theta_{l2}\right)$, the consumers who do not rent any durable goods.

The total utility of the consumers on the interval $\left[\theta_{l1},1\right]$ in period *t* is:

$$u_{\ln}^{t}\left(\theta \right)=\theta v-p_{\ln}+\rho u_{\ln}^{t+1}\left(\theta \right)$$

where $\theta v-p_{\ln}$ corresponds to the utility that consumers obtain from renting a new durable good; $u_{\ln}^{t+1}\left(\theta \right)$ corresponds to the utility that consumers obtain from renting a new durable goods in period t+1.

At the steady-state equilibrium, we observe that $u_{\ln}^{t}\left(\theta \right)=u_{\ln}^{t+1}\left(\theta \right)$. Therefore, we can obtain that the net utility of consumers on the interval $\left[\theta_{l1},1\right]$ in period *t* is:

(1) $$u_{\ln}^{t}\left(\theta \right)=\frac{\theta v-p_{\ln}}{1-\rho }$$

The total utility of the consumers on the interval $\left[\theta_{l2},\theta_{l1}\right)$ in period *t* is:

$$u_{\mathrm{lu}}^{t}\left(\theta \right)=\theta \delta v-p_{\mathrm{lu}}+\rho u_{\mathrm{lu}}^{t+1}\left(\theta \right)$$

At the steady-state equilibrium, we observe that $u_{\mathrm{lu}}^{t}\left(\theta \right)=u_{\mathrm{lu}}^{t+1}\left(\theta \right)$. Therefore, the net utility of the consumers on the interval $\left[\theta_{l2},\theta_{l1}\right)$ in period *t* is:

(2) $$u_{\mathrm{lu}}^{t}\left(\theta \right)=\frac{\theta \delta v-p_{\mathrm{lu}}}{1-\rho }$$

The total utility of the consumers on the interval $\left[0,\theta_{l2}\right)$ in period *t* is:

$$u_{l0}^{t}\left(\theta \right)=0$$

$\theta_{l1}$ corresponds to the marginal consumer who is indifferent between renting new goods and renting used goods, which means $u_{\ln}^{t}\left(\theta \right)=u_{\mathrm{lu}}^{t}\left(\theta \right)$. Hence, through (1) and (2), we have:

(3) $$\theta_{l1}=\frac{p_{\ln}-p_{\mathrm{lu}}}{\left(1-\delta \right)v}$$

$\theta_{l2}$ corresponds to the marginal consumer who is indifferent between renting used goods and not renting any goods at all.

Similar to the $\theta_{l1}$, we have:

(4) $$\theta_{l2}=\frac{p_{\mathrm{lu}}}{\delta v}$$

According to the consumer utility theory, we can derive the demand of the new and used durable goods in period *t* as follows:

(5) $$\{\begin{array}{c} q_{\ln}^{t}=1-\theta_{l1}\\ q_{\mathrm{lu}}^{t}=\theta_{l1}-\theta_{l2} \end{array}$$

Substituting (3) and (4) into (5), we can derive the price of leasing new and used goods as follows:

(6) $$\{\begin{array}{c} p_{\ln}=v\left(1-q_{\ln}^{t}-\delta q_{\mathrm{lu}}^{t}\right)\\ p_{\mathrm{lu}}=\delta v\left(1-q_{\ln}^{t}-q_{\mathrm{lu}}^{t}\right) \end{array}$$

According to the assumption, under the leasing strategy, carbon emissions in both the production and the consumption process are constrained by the carbon cap-and-trade policy. Hence, the profit function of the monopoly manufacturer in period *t* can be expressed as:

$$\pi_{l}^{t}=p_{\ln}q_{\ln}^{t}+p_{\mathrm{lu}}q_{\mathrm{lu}}^{t}-p_{e}\left(e_{0}q_{\ln}^{t}+e_{1}q_{\ln}^{t}+e_{2}q_{\mathrm{lu}}^{t}-Q\right)$$

According to the assumption, a new product will be rented out as a used product for the next period, this implies that $q_{\mathrm{lu}}^{t}=q_{\ln}^{t-1}$. Furthermore, at the steady-state equilibrium, we have $q_{\mathrm{lu}}^{t}=q_{\ln}^{t-1}=q_{\ln}^{t}$. Then, by substituting (6), the leasing profit function for the manufacturer is:

(7) $$\pi_{l}^{t}=\left(1+\delta \right)vq_{\ln}^{t}-\left(1+3\delta \right)vq_{\ln}^{t}{}^{2}-p_{e}\left(e_{0}+e_{1}+e_{2}\right)q_{\ln}^{t}+p_{e}Q$$

Through the second-order condition, we find that $\frac{\partial^{2}\pi_{l}^{t}}{\partial q_{\ln}^{t}{}^{2}}<0$. $\pi_{l}^{t}$ is concave function. Hence, the monopolist obtains the maximum profit by taking the first-order derivatives of Equation (7) with respect to $q_{\ln}^{t}$ and letting it be equal to 0. We can derive the optimal rental quantities of the new durable goods as follows:

(8) $$q_{\ln}^{t*}=\frac{\left(1+\delta \right)v-p_{e}\left(e_{0}+e_{1}+e_{2}\right)}{2\left(1+3\delta \right)v}$$

we assume $p_{e}<\frac{\left(1+\delta \right)v}{e_{0}+e_{1}+e_{2}}$ to ensure a positive profit.

Substituting Equations (8) in (6) and (7), we have the optimal leasing prices of the new and used durable goods, and the optimal leasing profit as follows:

(9) $$p_{\ln}^{*}=\frac{(-\delta^{2}+4\delta +1)v+\left(1+\delta \right)p_{e}\left(e_{0}+e_{1}+e_{2}\right)}{2\left(1+3\delta \right)}$$

(10) $$p_{lu}^{*}=\frac{2\delta^{2}v+\delta p_{e}\left(e_{0}+e_{1}+e_{2}\right)}{1+3\delta }$$

(11) $$\pi_{l}^{t*}=\frac{{\left[(1+\delta )v-p_{e}\left(e_{0}+e_{1}+e_{2}\right)\right]}^{2}}{4\left(1+3\delta \right)v}+p_{e}Q$$

### 4.2. Selling Model

Under the selling model, the monopolist only offers new durable goods through selling services. Therefore, we can partition consumers into three different groups: on the interval $\left[\theta_{s1},1\right]$, the consumers who only buy new durable goods in every period; on the interval $\left[\theta_{s2},\theta_{s1}\right)$, the consumers who only buy used durable goods in every period; and on the interval $\left[0,\theta_{s2}\right)$, the consumers who do not buy any durable goods. According to the assumption, the consumers own the product; therefore, the tax due from carbon emissions in the consumption process is borne by the consumers.

Therefore, the total utility of the consumers on the interval $\left[\theta_{s1},1\right]$ in period *t* is:

$$u_{sn}^{t}(\theta )=\theta v-p_{sn}-\lambda e_{1}+\rho p_{su}+\rho u_{sn}^{t+1}(\theta )$$

At the steady-state equilibrium, we observe that $u_{\mathrm{sn}}^{t}\left(\theta \right)=u_{\mathrm{sn}}^{t+1}\left(\theta \right)$. Therefore, the net utility of the consumers on the interval $\left[\theta_{s1},1\right]$ in period *t* is:

$$u_{sn}^{t}\left(\theta \right)=\frac{\theta v-p_{sn}-\lambda e_{1}+\rho p_{su}}{1-\rho }$$

The total utility of the consumers on the interval $\left[\theta_{s2},\theta_{s1}\right)$ in period *t* is:

$$u_{su}^{t}(\theta )=\theta \delta v-p_{su}-\lambda e_{2}+\rho u_{su}^{t+1}(\theta )$$

At the steady-state equilibrium, we observe that $u_{\mathrm{su}}^{t}\left(\theta \right)=u_{\mathrm{su}}^{t+1}\left(\theta \right)$. Therefore, the net utility of the consumers on the interval $\left[\theta_{s2},\theta_{s1}\right)$ in period t is:

$$u_{su}^{t}\left(\theta \right)=\frac{\theta \delta v-p_{su}-\lambda e_{2}}{1-\rho }$$

The total utility of the consumers on the interval $\left[0,\theta_{s2}\right)$ in period *t* is:

$$u_{s0}^{t}\left(\theta \right)=0$$

Similar to the leasing model, we have:

(12) $$\theta_{s1}=\frac{p_{sn}-\left(1+\rho \right)p_{su}+\lambda \left(e_{1}-e_{2}\right)}{\left(1-\delta \right)v}$$

(13) $$\theta_{s2}=\frac{p_{su}+\lambda e_{2}}{\delta v}$$

According to the assumption and the consumer utility theory, the demand of new and used durable goods in period *t* can be expressed as follows:

(14) $$\{\begin{array}{c} q_{sn}^{t}=1-\theta_{s1}\\ q_{su}^{t}=\theta_{s1}-\theta_{s2}=1-\theta_{s1} \end{array}$$

Substituting (12) and (13) into (14), we can derive the price of selling new product and the market clearing price of the used durable goods as follows:

(15) $$p_{sn}=\left(1+\rho \delta \right)v-\left(1+\delta +2\rho \delta \right)vq_{sn}^{t}-\lambda \left(e_{1}+\rho e_{2}\right)$$

(16) $$p_{su}=\frac{\left(\delta^{2}-\delta \right)v+2\delta p_{sn}+\lambda \left[2\delta e_{1}-\left(1+\delta \right)e_{2}\right]}{1+\delta +2\rho \delta }$$

According to the assumption, the carbon tax on carbon emissions during consumption is borne by consumers because they own the durable goods. The manufacturer covers the expenses of carbon emissions in the production process. Therefore, the profit function of the monopoly manufacturer in period *t* can be expressed as:

(17) $$\pi_{s}^{t}=p_{sn}q_{sn}^{t}-p_{e}\left(e_{0}q_{sn}^{t}-Q\right)$$

Through the second-order condition, we find that $\frac{\partial^{2}\pi_{s}^{t}}{\partial q_{sn}^{t}{}^{2}}<0$. $\pi_{s}^{t}$ is concave function. Hence, the monopolist has the maximum profit by taking the first-order derivatives of Equation (17) with respect to $q_{sn}^{t}$ and letting it be equal to 0. We can derive the optimum sales volume of new durable goods as follows:

(18) $$q_{sn}^{t*}=\frac{\left(1+\rho \delta \right)v-p_{e}e_{0}-\lambda \left(e_{1}+\rho e_{2}\right)}{2\left(1+\delta +2\rho \delta \right)v}$$

We assume $p_{e}<\frac{\left(1+\rho \delta \right)v-\lambda \left(e_{1}+\rho e_{2}\right)}{e_{0}}$ to ensure a positive profit.

Substituting Equations (18) in (15–17), we can derive the optimal selling price of the new durable goods, the market clearing price, and the optimal selling profit as follows:

(19) $$p_{sn}^{t*}=\frac{\left(1+\rho \delta \right)v+p_{e}e_{0}-\lambda \left(e_{1}+\rho e_{2}\right)}{2}$$

(20) $$p_{su}^{t*}=\frac{\left(1+\rho \right)\delta^{2}v+\delta p_{e}e_{0}+\delta \lambda e_{1}-\left(1+\delta +2\rho \delta \right)\lambda e_{2}}{1+\delta +2\rho \delta }$$

(21) $$\pi_{s}^{t*}=\frac{{\left[\left(1+\rho \delta \right)v-p_{e}e_{0}-\lambda \left(e_{1}+\rho e_{2}\right)\right]}^{2}}{4\left(1+\delta +2\rho \delta \right)v}+p_{e}Q$$

## 5. The Mixed Carbon Policy Impact Analysis

This section analyses how the manufacturer’s production quantity, the leasing and selling profits, and the consumer’s behaviour change after the mixed carbon policy is implemented.

### 5.1. The Impact of the Mixed Carbon Policy on Production

According to the Equations (8) and (18), using the necessary conditions for equilibrium, we can obtain the following results:

**Proposition** **** When $p_{e}>\max\{\frac{\left(1+\delta \right)v}{e_{0}+e_{1}+e_{2}},\frac{\left(1+\rho \delta \right)v-\lambda \left(e_{1}+\rho e_{2}\right)}{e_{0}}\}$, whether under leasing or selling, the manufacturer does not produce the durable goods, instead choosing to sell all the carbon quota Q. Only when $p_{e}<\min\{\frac{\left(1+\delta \right)v}{e_{0}+e_{1}+e_{2}},\frac{\left(1+\rho \delta \right)v-\lambda \left(e_{1}+\rho e_{2}\right)}{e_{0}}\}$, the manufacturer will carry on the production activity under leasing and selling. When $\min\{\frac{\left(1+\delta \right)v}{e_{0}+e_{1}+e_{2}},\frac{\left(1+\rho \delta \right)v-\lambda \left(e_{1}+\rho e_{2}\right)}{e_{0}}\}<p_{e}<\max\{\frac{\left(1+\delta \right)v}{e_{0}+e_{1}+e_{2}},\frac{\left(1+\rho \delta \right)v-\lambda \left(e_{1}+\rho e_{2}\right)}{e_{0}}\}$, depending on the level of the carbon tax rate, the manufacturer only carries on the production activity under one marketing model (leasing or selling).

**Proof.**  See Appendix A. □

Proposition 1 shows that the carbon trading price and the carbon tax rate have significant impacts on the manufacturer’s production decision. Different from the general assumption, the high carbon trading price is not always good for the policy-maker. The manufacturer’s incentive to produce decreases as the carbon trading price increases ($\frac{\partial q_{\ln}^{*}}{\partial p_{e}}<0,\frac{\partial q_{sn}^{*}}{\partial p_{e}}<0$). When the carbon trading price is too high, the manufacturer has no willingness to produce the durable goods because under this situation, the profit of selling all the carbon quota *Q* is higher than the profits obtained from leasing or selling the durable goods. Thus, to guarantee the effective implementation of the mixed carbon policy, policy-makers must keep the carbon trading price below the threshold ($p_{e}<\min\{\frac{\left(1+\delta \right)v}{e_{0}+e_{1}+e_{2}},\frac{\left(1+\rho \delta \right)v-\lambda \left(e_{1}+\rho e_{2}\right)}{e_{0}}\}$).

In addition, since the carbon tax is imposed on consumers, when the carbon tax rate is low ($\lambda <\frac{\left(1+\rho \delta \right)\left(e_{1}+e_{2}\right)-\left(1-\rho \right)\delta e_{0}}{\left(e_{1}+\rho e_{2}\right)\left(e_{0}+e_{1}+e_{2}\right)}v$), the manufacturer has a higher incentive to resume selling the durable goods, as the price of carbon trading price decreases. Additionally, when the carbon tax rate is high ($\lambda >\frac{\left(1+\rho \delta \right)\left(e_{1}+e_{2}\right)-\left(1-\rho \right)\delta e_{0}}{\left(e_{1}+\rho e_{2}\right)\left(e_{0}+e_{1}+e_{2}\right)}v$), the manufacturer has a higher incentive to resume leasing the durable goods, as the price of the carbon trading price decreases. According to our assumptions, carbon cap-and-trade is more effective at controlling total carbon emissions; thus, holding the carbon tax to a high level ($\lambda >\frac{\left(1+\rho \delta \right)\left(e_{1}+e_{2}\right)-\left(1-\rho \right)\delta e_{0}}{\left(e_{1}+\rho e_{2}\right)\left(e_{0}+e_{1}+e_{2}\right)}v$) would be more beneficial for policymakers in controlling the total amount of carbon emissions.

### 5.2. The Impact of the Mixed Carbon Policy on Consuming Behaviour

#### 5.2.1. Leasing Situation

At the steady-state equilibrium, under the mixed carbon policy, substituting Equations (9) and (10) into Equations (3) and (4), we can derive the indifference utility value of $\theta_{l}^{*}$ as follows:

(22) $$\theta_{l1}^{*}=\frac{(1+5\delta )v+p_{e}\left(e_{0}+e_{1}+e_{2}\right)}{2\left(1+3\delta \right)v}$$

(23) $$\theta_{l2}^{*}=\frac{2\delta v+p_{e}\left(e_{0}+e_{1}+e_{2}\right)}{\left(1+3\delta \right)v}$$

Before the implementation of the mixed carbon policy, $p_{e}=0$. Substituting $p_{e}=0$ into Equations (22) and (23), we can derive the indifference utility value of $\theta_{l}^{\prime *}$ as follows:

(24) $$\theta_{l1}^{\prime *}=\frac{1+5\delta }{2\left(1+3\delta \right)}$$

(25) $$\theta_{l2}^{\prime *}=\frac{2\delta }{1+3\delta }$$

According to Equation (5) and by combining Equations (22–25), we can derive the leasing reduction quantities of new products, used products and total products under the mixed carbon policy as follows:

$$\Delta q_{\ln}^{*}=\left(1-\theta_{l1}^{\prime *}\right)-\left(1-\theta_{l1}^{*}\right)=\frac{p_{e}\left(e_{0}+e_{1}+e_{2}\right)}{2\left(1+3\delta \right)v}>0$$

$$\Delta q_{lu}^{*}=\left(\theta_{l1}^{\prime *}-\theta_{l2}^{\prime *}\right)-\left(\theta_{l1}^{*}-\theta_{l2}^{*}\right)=\frac{p_{e}\left(e_{0}+e_{1}+e_{2}\right)}{2\left(1+3\delta \right)v}>0$$

$$\Delta q_{l}^{*}=\left(1-\theta_{l2}^{\prime *}\right)-\left(1-\theta_{l2}^{*}\right)=\frac{p_{e}\left(e_{0}+e_{1}+e_{2}\right)}{\left(1+3\delta \right)v}>0$$

where $\Delta q_{\ln}^{*}$ is the reduction in the quantity of the consumers who always rent the new products. Under the mixed carbon policy, this group of consumers turns to renting the used products instead. $\Delta q_{lu}^{*}$ is the reduction in the quantity of consumers who always rent the used products; this group of consumers withdraws from the market and no longer rents any of the durable goods. Under the mixed carbon policy, $\Delta q_{l}^{*}$ is the total reduction in the quantity of consumers who rent the durable goods. Hence, the following is proposed:

**Proposition** **2.** The total number of consumers renting durable goods decreases with increases in the carbon trading price. In addition, the number of consumers renting new durable goods is decreasing at the same rate as that of the decrease in the number of consumers renting used durable goods.

**Proof.**  See Appendix B. □

Proposition 2 shows that the carbon trading price has a significant impact on the consumers’ consumption behaviour under a leasing strategy. The implementation of the mixed carbon policy has led to a reduction in the number of consumers renting old and new durable goods, and the reduction number is the same. The reduction in the number of consumers renting old and new durable goods increases as the carbon trading price increases. In addition, the higher the carbon emissions of the durable goods in the production and the consumption process are, the greater the reduction in the number of the consumers. In addition, the quality *v* and the durability *δ* of the durable goods also affect the consumption behaviours under leasing: the higher the value of *v* and *δ* are, the less the reduction in the number of consumers.

#### 5.2.2. Selling Situation

Similar to the leasing situation, we can derive the indifference utility value of $\theta_{s}^{*}$ with the mixed carbon policy as follows:

(26) $$\theta_{s1}^{*}=\frac{(1+2\delta +3\rho \delta )v+p_{e}e_{0}+\lambda \left(e_{1}+\rho e_{2}\right)}{2\left(1+\delta +2\rho \delta \right)v}$$

(27) $$\theta_{s2}^{*}=\frac{(1+\rho )\delta v+p_{e}e_{0}+\lambda \left(e_{1}+\rho e_{2}\right)}{\left(1+\delta +2\rho \delta \right)v}$$

Before the implementation of the mixed carbon policy, $p_{e}=\lambda =0$. Substituting $p_{e}=\lambda =0$ into Equations (26) and (27), we can derive the indifference utility value of $\theta_{s}^{\prime *}$ without the carbon cap-and-trade policy and the carbon tax policy as follows:

(28) $$\theta_{s1}^{\prime *}=\frac{1+2\delta +3\rho \delta }{2\left(1+\delta +2\rho \delta \right)}$$

(29) $$\theta_{s2}^{*}=\frac{(1+\rho )\delta }{1+\delta +2\rho \delta }$$

According to Equation (14) and by combining Equations (26–29), we can derive the selling reduction quantities of the new products, the used products and the total products when the government enacts a carbon cap-and-trade policy and a carbon tax policy as follows:

$$\Delta q_{sn}^{*}=\left(1-\theta_{s1}^{\prime *}\right)-\left(1-\theta_{s1}^{*}\right)=\frac{p_{e}e_{0}+\lambda \left(e_{1}+\rho e_{2}\right)}{2\left(1+\delta +2\rho \delta \right)v}>0$$

$$\Delta q_{su}^{*}=\left(\theta_{s1}^{\prime *}-\theta_{s2}^{\prime *}\right)-\left(\theta_{s1}^{*}-\theta_{s2}^{*}\right)=\frac{p_{e}e_{0}+\lambda \left(e_{1}+\rho e_{2}\right)}{2\left(1+\delta +2\rho \delta \right)v}>0$$

$$\Delta q_{s}^{*}=\left(1-\theta_{s2}^{\prime *}\right)-\left(1-\theta_{s2}^{*}\right)=\frac{p_{e}e_{0}+\lambda \left(e_{1}+\rho e_{2}\right)}{\left(1+\delta +2\rho \delta \right)v}>0$$

where $\Delta q_{sn}^{*}$ is the reduction in the quantity of consumers who always buy new products. Under the mixed carbon policy, this group of consumers turns to buy used products instead. $\Delta q_{su}^{*}$ is the reduction in the quantity of consumers who always buy used products; this group of consumers withdraws from the market and no longer buys any products. Under the mixed carbon policy, $\Delta q_{s}^{*}$ is the total reduction in the quantity of consumers who buy the durable goods. Hence, the following is proposed:

**Proposition** **3.** The total number of consumers buying durable goods decreases with the increases of the carbon trading price and the carbon tax rate. In addition, the number of consumers buying new durable goods is decreasing at the same rate as the rate of the decrease in the number of consumers buying used durable goods. Compared with the carbon trading price, the carbon tax has a greater impact on consumption behaviours.

**Proof.**  See Appendix C. □

Proposition 3 shows that the carbon trading price and the carbon tax rate have significant impacts on the consumers’ consumption behaviours under a selling strategy. The implementation of the mixed carbon policy has led to a reduction in the number of consumers buying old and new durable goods, and the reduction in the numbers is the same. The reduction in the number of consumers buying old and new durable goods increases as the carbon trading price and carbon tax rate increase. In addition, the higher the carbon emissions of the durable goods in the production and the consumption process are, the more the reduction in the number of consumers. In addition, due to the durable goods’ higher carbon emissions in the consumption process, the carbon tax rate has a greater impact on the consumers’ consumption behaviour. Therefore, the carbon tax is a better carbon policy mechanism that the government can employ to regulate and control the consumption behaviours.

Together with the results of Proposition 2 and Proposition 3, we find that the influencing characteristics of the mixed carbon policy on the consumers’ consumption behaviours under leasing and selling are similar. The number of consumers participating in the market both decreases, and the decreasing rate is the same. Moreover, in regulating consumer behaviours, a carbon tax is more effective than a carbon price.

### 5.3. The Impact of the mixed Carbon Policy on Profits

From Section 5.2, we know that the number of consumers participating in the market decreases after the government enacts the mixed carbon policy. However, does that mean the manufacturer’s profits also decline? In this section, we study how the mixed carbon policy affects the manufacturer’s leasing and selling profits.

#### 5.3.1. Leasing Situation

Comparing the leasing profits before and after the mixed carbon policy has been enacted, assume the government does not enact the mixed carbon policy, that is, assume that $p_{e}=0$. Substituting $p_{e}=0$ into Equation (11), without a mixed carbon policy, we can derive the optimal profits of the manufacturer as follows:

$$\pi_{l}^{\prime *}=\frac{{\left(1+\delta \right)}^{2}v}{4\left(1+3\delta \right)}$$

where $\pi_{l}^{\prime *}$ denotes the optimal leasing profits with no mixed carbon policy.

By comparing the profit difference for the manufacturer between $\pi_{l}^{t*}$ and $\pi_{l}^{\prime *}$, we have:

(30) $$\Delta \pi_{l}=\pi_{l}^{\prime *}-\pi_{l}^{t*}=\frac{2(1+\delta )v-p_{e}\left(e_{0}+e_{1}+e_{2}\right)}{4\left(1+3\delta \right)v}p_{e}\left(e_{0}+e_{1}+e_{2}\right)-p_{e}Q$$

Taking the first-order derivatives of $\Delta \pi_{l}$ with respect to $p_{e}$, we then have:

(31) $$\frac{\partial \Delta \pi_{l}}{\partial p_{e}}=\frac{(1+\delta )v-p_{e}\left(e_{0}+e_{1}+e_{2}\right)}{2\left(1+3\delta \right)v}\left(e_{0}+e_{1}+e_{2}\right)-Q$$

**Proposition** **4.** *The following result holds:*

*(1)*  When $Q<\left(e_{0}+e_{1}+e_{2}\right)q_{\ln}^{t*}$, we have $\Delta \pi_{l}>0$, $\frac{\partial \Delta \pi_{l}}{\partial p_{e}}>0$: the optimal leasing profit declined after the enactment of the mixed carbon policy. The higher the carbon trading price is, the lower the leasing profit.
*(2)*  When $\left(e_{0}+e_{1}+e_{2}\right)q_{\ln}^{t*}<Q<Q_{A}$, we have $\Delta \pi_{l}>0$, $\frac{\partial \Delta \pi_{l}}{\partial p_{e}}<0$: the optimal leasing profit still declined after the enactment of the mixed carbon policy, but the higher the carbon trading price is, the less the lost leasing profit.
*(3)*  *When $Q>Q_{A}$, we have $\Delta \pi_{l}<0$, $\frac{\partial \Delta \pi_{l}}{\partial p_{e}}<0$: the optimal leasing profit under the mixed carbon policy is higher than that without the mixed carbon policy, and the higher the carbon trading price is, the greater the leasing profit.*

Note: $e_{0}+e_{1}+e_{2}$ denotes the total carbon emissions of one durable product in the whole life cycle; thereby, $\left(e_{0}+e_{1}+e_{2}\right)q_{\ln}^{t*}$ denotes the total carbon emissions of all leased durable goods. We assume that $Q_{A}=\left(e_{0}+e_{1}+e_{2}\right)q_{\ln}^{t*}+\frac{p_{e}{\left(e_{0}+e_{1}+e_{2}\right)}^{2}}{4\left(1+3\delta \right)v}$.

**Proof.**  See Appendix D. □

Proposition 4 shows that when the government sets the value of the carbon cap *Q* too low (i.e., lower than the total actual carbon emissions from the manufacturer $\left(e_{0}+e_{1}+e_{2}\right)q_{\ln}^{t*}$), the manufacturer’s leasing profits are badly dented. Additionally, as the carbon trading price increases, the manufacturer’s leasing profits will be further eroded. With the increase of carbon cap *Q*, the profit level of the manufacturer gradually improves, when $\left(e_{0}+e_{1}+e_{2}\right)q_{\ln}^{t*}<Q<Q_{A}$; although the mixed carbon policy still reduces the manufacturer’s leasing profits, the manufacturer can already obtain a benefit by selling excess carbon cap permits. Moreover, the higher the carbon trading price is, the less the leasing profits are reduced. When the carbon cap *Q* is too high ($Q>Q_{A}$), the profits than manufacturer can earn by selling excess carbon cap permits is more than the market shrinkage caused by the mixed carbon policy. Thereby, the manufacturer’s leasing profit is higher than it was before the mixed carbon policy was implemented, and the higher the carbon trading price is, the more the manufacturer’s profit will increase.

As seen from the above analysis, a carbon cap *Q* that is too low will severely decrease the manufacturer’s incentive to produce; however, the government’s goal of controlling carbon emissions will not be achieved if the carbon cap *Q* is too high. By setting the carbon cap *Q* on the interval $\left[\left(e_{0}+e_{1}+e_{2}\right)q_{\ln}^{t*},Q_{A}\right]$, the manufacturer can be encouraged to reduce the carbon emissions, while not overly affecting the incentives for production.

#### 5.3.2. Selling Situation

Similar to the leasing situation, before the government enacts the mixed carbon policy, $p_{e}=\lambda =0$. Substituting $p_{e}=\lambda =0$ into Equation (21), without the mixed carbon policy, the optimal selling profit of the manufacturer can be derived as follows:

$$\pi_{s}^{\prime *}=\frac{{\left(1+\rho \delta \right)}^{2}v}{4\left(1+\delta +2\rho \delta \right)}$$

where $\pi_{s}^{\prime *}$ denotes the optimal selling profits without the mixed carbon policy.

By comparing the profit difference for the manufacturer between $\pi_{s}^{t*}$ and $\pi_{s}^{\prime *}$, we have:

(32) $$\Delta \pi_{s}=\pi_{s}^{\prime *}-\pi_{s}^{t*}=\frac{2\left(1+\rho \delta \right)v-p_{e}e_{0}-\lambda \left(e_{1}+\rho e_{2}\right)}{4\left(1+\delta +2\rho \delta \right)v}\left(p_{e}e_{0}+\lambda e_{1}+\rho \lambda e_{2}\right)-p_{e}Q$$

Taking the first-order derivatives of $\Delta \pi_{l}$ with respect to $p_{e}$ and λ, then, we have:

(33) $$\frac{\partial \Delta \pi_{s}}{\partial p_{e}}=\frac{\left(1+\rho \delta \right)v-p_{e}e_{0}-\lambda \left(e_{1}+\rho e_{2}\right)}{2\left(1+\delta +2\rho \delta \right)v}e_{0}-Q$$

(34) $$\frac{\partial \Delta \pi_{s}}{\partial \lambda }=\frac{\left(1+\rho \delta \right)v-p_{e}e_{0}-\lambda \left(e_{1}+\rho e_{2}\right)}{2\left(1+\delta +2\rho \delta \right)v}\left(e_{1}+\rho e_{2}\right)$$

**Proposition** **5.** *The optimal selling profits of the manufacturer decrease as the carbon tax rate λ increases. Note the following for the carbon cap Q and the carbon trading price $p_{e}$:*

*(1)*  When $Q<e_{0}q_{sn}^{t*}$, we have $\Delta \pi_{s}>0$, $\frac{\partial \Delta \pi_{s}}{\partial p_{e}}>0$: the optimal selling profit declined after the enactment of the mixed carbon policy, and the higher the carbon trading price is, the lower the selling profit.
*(2)*  When $e_{0}q_{sn}^{t*}<Q<Q_{B}$, we have $\Delta \pi_{s}>0$, $\frac{\partial \Delta \pi_{s}}{\partial p_{e}}<0$: the optimal selling profit still declined after the enactment of the mixed carbon policy, but the higher the carbon trading price is, the less the lost selling profit.
*(3)*  *When $Q>Q_{B}$, we have $\Delta \pi_{s}<0$, $\frac{\partial \Delta \pi_{s}}{\partial p_{e}}<0$: the optimal selling profit under the mixed carbon policy is higher than that without the mixed carbon policy, and the higher the carbon trading price is, the more the selling profit.*

Note: $e_{0}q_{sn}^{t*}$ denotes the carbon emissions of all selling products in the production process. $\left(\lambda e_{1}+\rho \lambda e_{2}\right)q_{sn}^{t*}$ denotes the total carbon tax of all selling products during the consumption process. We assume that $Q_{B}=\frac{\left(p_{e}e_{0}+\lambda e_{1}+\rho \lambda e_{2}\right)q_{sn}^{t*}}{p_{e}}+\frac{{\left(p_{e}e_{0}+\lambda e_{1}+\rho \lambda e_{2}\right)}^{2}}{4\left(1+\delta +2\rho \delta \right)vp_{e}}$.

**Proof.**  See Appendix E. □

Compared with the results of Proposition 4, we find that the impact of the carbon cap *Q* on leasing and selling is very similar. When the carbon cap *Q* is lower than the total actual carbon emissions from the manufacturer, that is, when $Q<e_{0}q_{sn}^{t*}$, the manufacturer’s selling profits are lower than they were before the mixed carbon policy was implemented, and as the carbon trading price increases, the selling profits fall further. When $e_{0}q_{sn}^{t*}<Q<Q_{B}$, the manufacturer’s selling profits are still lower than they were before the mixed carbon policy was implemented, but the higher the carbon trading price is, the less the leasing profits are reduced. When $Q>Q_{B}$, the manufacturer’s selling profits are higher than they were before the mixed carbon policy was implemented, and the higher the carbon trading price is, the more the selling profits increase. Therefore, similar to the leasing situation, by setting the carbon cap *Q* on the interval $\left[e_{0}q_{sn}^{t*},Q_{B}\right]$, the manufacturer can be encouraged to reduce the carbon emissions, while not overly affecting the manufacturer’s incentives for production.

Together with the results of Proposition 4 and Proposition 5, we find that the manufacturer’s leasing and selling profits are both affected by the mixed carbon policy. For the carbon tax rate, the higher the carbon tax rate is, the lower the manufacturer’s profits. The carbon cap and carbon trading price combine to affect the manufacturer’s profits. When the carbon cap *Q* is low, raising the carbon trading price will hurt the manufacturer’s profits; when the carbon cap *Q* is high, raising the carbon trading price will boost the manufacturer’s profits. Therefore, between the government and the manufacture, for the carbon cap *Q*, there is an equilibrium interval ($\{\begin{array}{ll} \left[\left(e_{0}+e_{1}+e_{2}\right)q_{\ln}^{t*},Q_{A}\right] & \mathrm{under}\;\mathrm{leasing}\\ \left[e_{0}q_{sn}^{t*},Q_{B}\right] & \mathrm{under}\;\mathrm{selling} \end{array}$) in which the government can achieve the aim of controlling carbon emissions, while not overly affecting the manufacturer’s enthusiasm for production.

### 5.4. Optimal Marketing Model Selection

After the mixed carbon policy was enacted, the manufacturer’s optimal marketing model selection changed. Leasing is no longer the optimal marketing model all the time. In this case, this section studies how the mixed carbon policy affects the manufacturer’s optimal marketing model.

Comparing the leasing and selling profits of the manufacturer under the mixed carbon policy, according to the Equations (11) and (21), we can obtain the following results:

**Proposition** **6.** When the carbon trading price $p_{e}=p_{e}^{*}$, $\pi_{l}^{t*}=\pi_{s}^{t*}$, i.e., when the manufacturer’s leasing and selling profits are same, there is no difference between the leasing and the selling model; when $0<p_{e}<p_{e}^{*}$, $\pi_{l}^{t*}>\pi_{s}^{t*}$; therefore, leasing is the optimal marketing model; when $p_{e}>p_{e}^{*}$, $\pi_{l}^{t*}<\pi_{s}^{t*}$; therefore, selling is the optimal marketing model.

Note: let $p_{e}^{*}=\frac{\left[(1+\delta )\sqrt{1+\delta +2\rho \delta }-(1+\rho \delta )\sqrt{1+3\delta }\right]v+\sqrt{1+3\delta }(e_{1}+\rho e_{2})\lambda }{\sqrt{1+\delta +2\rho \delta }(e_{0}+e_{1}+e_{2})-\sqrt{1+3\delta }e_{0}}$.

**Proof.**  See Appendix F. □

Proposition 6 shows that the carbon tax rate cannot affect the marketing model selection of the manufacturer. In contrast, the carbon trading price has a significant impact on the market model selection of the manufacturer. When the carbon trading price is low, the manufacturer is more inclined to choose leasing as its optimal marketing model; when the carbon trading price is high, the manufacturer is more inclined to choose selling as its optimal marketing model.

Together with the results of Proposition 4 and Proposition 5, we find that when the carbon trading price $0<p_{e}<p_{e}^{*}$, the manufacturer will choose the leasing model in order to maximize the profits; under these circumstances, to effectively control the overall carbon emissions, the government should set the carbon cap *Q* on the interval $\left[\left(e_{0}+e_{1}+e_{2}\right)q_{\ln}^{t*},Q_{A}\right]$. When the carbon trading price $p_{e}>p_{e}^{*}$, the manufacturer will choose the selling model in order to maximize the profits; under the circumstances, to effectively control the overall carbon emissions, the government should set the carbon cap *Q* on the interval $\left[e_{0}q_{sn}^{t*},Q_{B}\right]$.

### 5.5. Comparing Total Carbon Emissions under Leasing and Selling

We now compare the total actual carbon emissions of leasing and selling. According to the assumption, we can derive that the total carbon emissions of one durable good during the whole life cycle are always $e_{0}+e_{1}+e_{2}$. Therefore, $E_{l}=\left(e_{0}+e_{1}+e_{2}\right)q_{\ln}^{t*}$ denotes the actual total carbon emissions under leasing, and the actual total carbon emissions of selling are denoted by $E_{s}=\left(e_{0}+e_{1}+e_{2}\right)q_{sn}^{t*}$.

According to the Equations (8) and (18), we can obtain the following results:

**Proposition** **7.** *The following result holds:*

*(1)*  If the carbon tax rate $\lambda <\frac{\left[\left(1+3\delta \right)\left(1+\rho \delta \right)-\left(1+\delta +2\rho \delta \right)\left(1+\delta \right)\right]v}{\left(1+3\delta \right)(e_{1}+\rho e_{2})}$, we have $E_{l}<E_{s}$.
*(1)*  If the carbon tax rate $\lambda >\frac{\left[\left(1+3\delta \right)\left(1+\rho \delta \right)-\left(1+\delta +2\rho \delta \right)\left(1+\delta \right)\right]v}{\left(1+3\delta \right)(e_{1}+\rho e_{2})}$: When $p_{e}=p_{e}^{**}$, we have $E_{l}=E_{s}$; when $p_{e}<p_{e}^{**}$, we have $E_{l}>E_{s}$; and when $p_{e}>p_{e}^{**}$, we have $E_{l}<E_{s}$.

Note: let $p_{e}^{**}=\frac{\left(1+3\delta \right)(e_{1}+\rho e_{2})\lambda -\left[\left(1+3\delta \right)\left(1+\rho \delta \right)-\left(1+\delta +2\rho \delta \right)\left(1+\delta \right)\right]v}{\left(1+\delta +2\rho \delta \right)(e_{0}+e_{1}+e_{2})-\left(1+3\delta \right)e_{0}}$.

**Proof.**  See Appendix G. □

Proposition 7 shows that, when the carbon tax rate *λ* is low ($\lambda <\frac{\left[\left(1+3\delta \right)\left(1+\rho \delta \right)-\left(1+\delta +2\rho \delta \right)\left(1+\delta \right)\right]v}{\left(1+3\delta \right)(e_{1}+\rho e_{2})}$), the total carbon emissions under leasing are always lower than the total carbon emissions under selling are, regardless of how the carbon trading price changes. Hence, to encourage the manufacturer to choose the leasing model, the government should set the carbon cap *Q* on the interval $\left[\left(e_{0}+e_{1}+e_{2}\right)q_{\ln}^{t*},Q_{A}\right]$ and keep the carbon trading price lower than $p_{e}^{*}$, according to the Proposition 6. When the carbon tax rate *λ* is high ($\lambda >\frac{\left[\left(1+3\delta \right)\left(1+\rho \delta \right)-\left(1+\delta +2\rho \delta \right)\left(1+\delta \right)\right]v}{\left(1+3\delta \right)(e_{1}+\rho e_{2})}$) and the carbon trading price is lower than $p_{e}^{**}$, the total carbon emissions under leasing are higher than the total carbon emissions under selling. Hence, according to the Proposition 6, to encourage the manufacturer to choose the selling model, the government should set the carbon cap *Q* on the interval $\left[e_{0}q_{sn}^{t*},Q_{B}\right]$ and keep the carbon trading price higher than $p_{e}^{*}$; the total carbon emissions under leasing are lower than the total carbon emissions under selling when the carbon trading price is higher than $p_{e}^{**}$. Hence, to encourage the manufacturer to choose the leasing model, the government should set the carbon cap *Q* on the interval $\left[\left(e_{0}+e_{1}+e_{2}\right)q_{\ln}^{t*},Q_{A}\right]$ and keep the carbon trading price lower than $p_{e}^{*}$, according to the Proposition 6.

### 5.6. Win-Win Strategy

From the results of Section 5.3 and Section 5.4, we know that there exists a threshold value of carbon trading prices that makes the profits and the total actual carbon emissions under leasing the same as those under selling. Therefore, is there a carbon trading price range where the manufacturer gets higher profits and at the same time the total carbon emissions are lower? This section explores the existence of the intervals for the carbon trading price that achieve win-win results for the manufacturer and the environment.

According to the results of Proposition 6 and Proposition 7, we can obtain the following results:

**Proposition** **8.** *The following result holds:*

*(1)*  If the carbon tax rate $\lambda <\frac{\left[\left(1+3\delta \right)\left(1+\rho \delta \right)-\left(1+\delta +2\rho \delta \right)\left(1+\delta \right)\right]v}{\left(1+3\delta \right)(e_{1}+\rho e_{2})}$, we have: $\{\begin{array}{c} \pi_{l}^{t*}>\pi_{s}^{t*},E_{l}<E_{s}\mathrm{when}\;p_{e}<p_{e}^{*}\\ \pi_{l}^{t*}<\pi_{s}^{t*},E_{l}<E_{s}\mathrm{when}\;p_{e}>p_{e}^{*} \end{array}$.
*(2)*  If the carbon tax rate $\lambda >\frac{\left[\left(1+3\delta \right)\left(1+\rho \delta \right)-\left(1+\delta +2\rho \delta \right)\left(1+\delta \right)\right]v}{\left(1+3\delta \right)(e_{1}+\rho e_{2})}$, we have: $\{\begin{array}{c} \pi_{l}^{t*}>\pi_{s}^{t*},E_{l}>E_{s}\mathrm{when}\;p_{e}<p_{e}^{**}\\ \pi_{l}^{t*}>\pi_{s}^{t*},E_{l}<E_{s}\mathrm{when}\;p_{e}^{**}<p_{e}<p_{e}^{*}\;\\ \pi_{l}^{t*}<\pi_{s}^{t*},E_{l}<E_{s}\mathrm{when}\;p_{e}>p_{e}^{*} \end{array}$.

**Proof.**  See Appendix H. □

Proposition 8 shows that if the carbon tax rate is low ($\lambda <\frac{\left[\left(1+3\delta \right)\left(1+\rho \delta \right)-\left(1+\delta +2\rho \delta \right)\left(1+\delta \right)\right]v}{\left(1+3\delta \right)(e_{1}+\rho e_{2})}$), then, when the carbon trading price is high ($p_{e}>p_{e}^{*}$), the profits of the manufacturer under selling are higher than those under leasing, but the total carbon emissions under selling are also higher than those under leasing. When the carbon trading price is low ($p_{e}<p_{e}^{*}$), the profits of the manufacturer under leasing are higher than the selling profits are, and the total carbon emissions under leasing are meanwhile also lower than those under selling. Thereby, $\{\begin{array}{c} p_{e}<p_{e}^{*}\\ \lambda <\frac{\left[\left(1+3\delta \right)\left(1+\rho \delta \right)-\left(1+\delta +2\rho \delta \right)\left(1+\delta \right)\right]v}{\left(1+3\delta \right)(e_{1}+\rho e_{2})} \end{array}$ is an optimal equilibrium interval for the carbon trading price and the carbon tax rate that creates a win-win result between the manufacturer’s profits and a friendly environment.

If carbon tax rate is high ($\lambda >\frac{\left[\left(1+3\delta \right)\left(1+\rho \delta \right)-\left(1+\delta +2\rho \delta \right)\left(1+\delta \right)\right]v}{\left(1+3\delta \right)(e_{1}+\rho e_{2})}$), then, when the carbon trading price is too low ($p_{e}<p_{e}^{**}$), the profits of the manufacturer under leasing are higher than the selling profits, but the total carbon emissions under leasing are also higher than those under selling. When the carbon trading price is too high ($p_{e}>p_{e}^{*}$), the profits of the manufacturer under selling are higher than the leasing profits, but the total carbon emissions under selling are also higher than those under leasing. Only when the carbon trading price is on the interval $p_{e}^{**}<p_{e}<p_{e}^{*}$, the profits of the manufacturer under leasing are higher than selling profits, and at the same time, the total carbon emissions under leasing are also lower than those under selling. Hence, $\{\begin{array}{c} p_{e}^{**}<p_{e}<p_{e}^{*}\\ \lambda >\frac{\left[\left(1+3\delta \right)\left(1+\rho \delta \right)-\left(1+\delta +2\rho \delta \right)\left(1+\delta \right)\right]v}{\left(1+3\delta \right)(e_{1}+\rho e_{2})} \end{array}$ is another optimal equilibrium interval for the carbon trading price and carbon tax rate that creates a win-win result between the manufacturer’s profits and a friendly environment.

## 6. Numerical Analysis

To capture qualitative insight regarding how the manufacturer’s production and profits varies as the mixed carbon policy varies, in this section, we use a numerical analysis to further illustrate the impacts of the carbon trading price, carbon cap and carbon tax rate on the production quantities, consumption behaviour, the leasing and selling profits and the total carbon emissions. In our numerical analysis, we use the following values to establish ranges for model parameters: *δ* = 0.5, *v* = 1, ρ=0.6, $e_{0}=0.3$, $e_{1}=0.3$, $e_{2}=0.4$, $Q_{1}=0.15$, $Q_{2}=0.25$, $Q_{3}=0.35$, $Q_{4}=0.06$, $Q_{5}=0.08$, $Q_{6}=0.1$, $\lambda_{1}=0.1$, $\lambda_{2}=0.2$, $\lambda_{3}=0.3$. We draw the relationships in the following figures.

**Figure 1:**
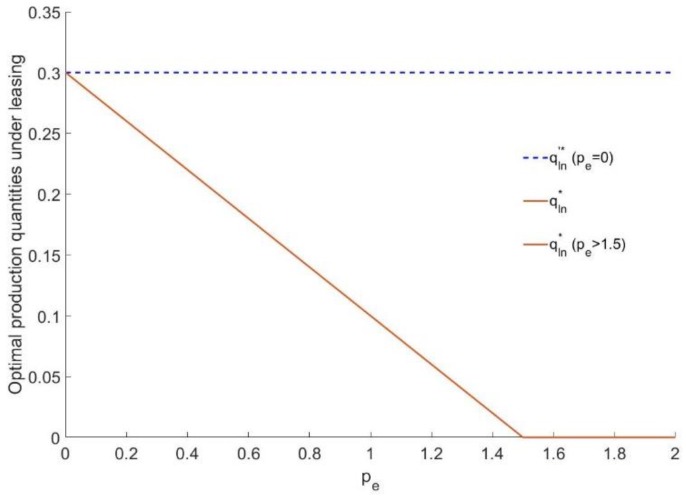
The relationship between the production quantities of the manufacturer and $p_{e}$ under a leasing strategy.

Figure 1 shows the effect of $p_{e}$ (carbon trading price) on the production quantity of the manufacturer under a leasing strategy. It is obvious that if the carbon policy is not implemented, the production quantity of the manufacturer under leasing is a fixed value $q_{\ln}^{\prime *}=0.3$. 

If the carbon policy is implemented, the optimal production quantity $q_{\ln}^{*}$ under leasing decreases as $p_{e}$ increases, and it remains 0 when $p_{e}\geq 1.5$. Under this circumstance, the manufacturer no longer makes the durable goods but sells the entire carbon emission cap permits.

**Figure 2:**
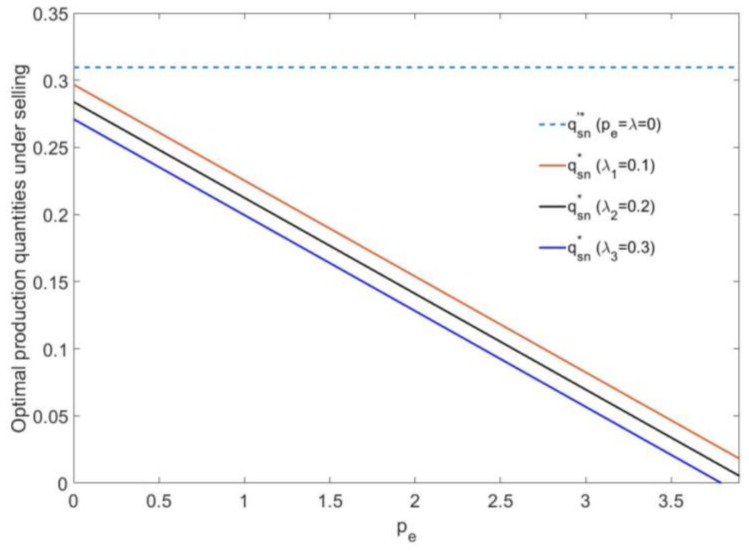
The relationship between the production quantities of the manufacturer and $p_{e}$ under a selling strategy.

In Figure 2, if the mixed carbon policy is not implemented ($p_{e}=\lambda =0$), the production quantity of the manufacturer under selling is a fixed value $q_{sn}^{\prime *}=0.31$. If the mixed carbon policy is implemented, the optimal production quantity $q_{sn}^{*}$ under selling decreases as $p_{e}$ increases, and the carbon tax rate does not affect the descending slope of the production quantity. The smaller the carbon tax rate *λ* is, the closer the production quantity is to 0.31. Whatever the carbon tax rate value is, the manufacturer will no longer make any durable goods when the carbon trading price is large enough.

**Figure 3:**
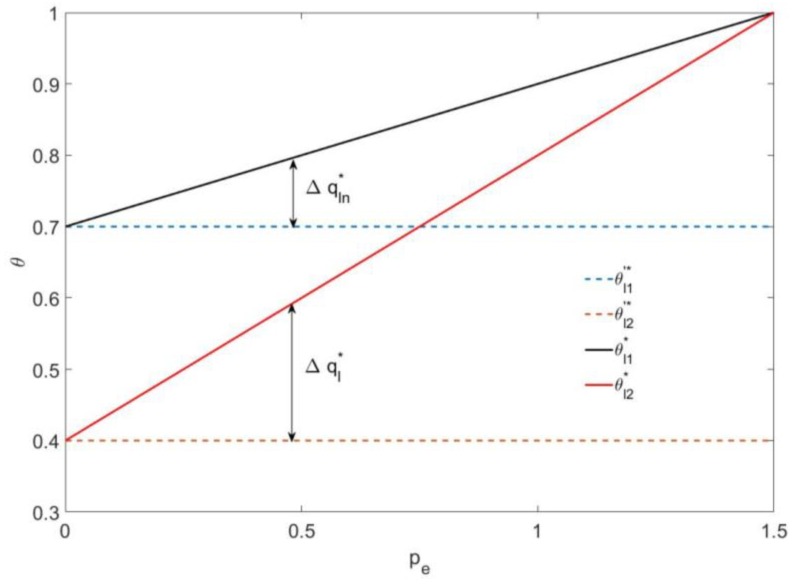
The effect of $p_{e}$ on the utility type of consumers under a leasing strategy.

As shown in Figure 3, if the mixed carbon policy is not implemented, the utility type value of the consumers who rent new durable goods and used durable goods are 0.7 and 0.4, respectively. If the mixed carbon policy is implemented, the utility type values of both types of consumers increase as the carbon trading price $p_{e}$ increases, which means that with the increases of the carbon trading price, both the number of consumers that rent new durable goods and those that rent used durable goods are reduced.

Additionally, the value of $\theta_{l2}^{*}$ is increasing at twice the rate of increase for $\theta_{l1}^{*}$ until $\theta_{l1}^{*}=\theta_{l2}^{*}=1$ when $p_{e}=1.5$, which means the reduction in the number of consumers who rent new durable goods $\Delta q_{\ln}^{*}$ is half the total reduction in the number of consumers $\Delta q_{l}^{*}$ and that the reduction in the number of consumers who rent new durable goods is equal to the reduction in the number of consumers who rent used durable goods. Furthermore, the leasing consumers will all exit the market when $p_{e}\geq 1.5$.

**Figure 4:**
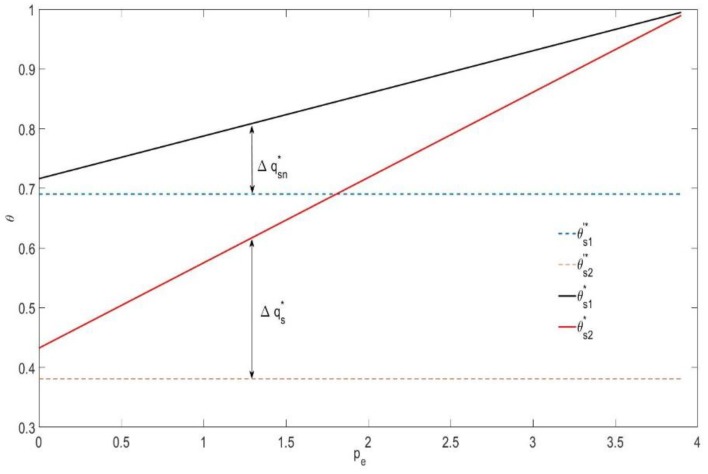
The effect of $p_{e}$ on the utility type of consumers under a selling strategy ($\lambda_{2}=0.2$ ).

In Figure 4, if the mixed carbon policy is not implemented, the utility type value of consumers who buy new durable goods and used durable goods are 0.45 and 0.38, respectively. Similar to the leasing strategy, if the mixed carbon policy is implemented, both types of consumers, i.e., those who buy new durable goods and those that buy used durable goods, are reduced with increases of the carbon trading price $p_{e}$. However, due to the existence of a carbon tax, the utility type value of consumers does not start at the value of the utility type without the carbon policy. Additionally, similar to leasing, the value of $\theta_{s2}^{*}$ is also increasing at twice the rate of increase of that for $\theta_{s1}^{*}$, i.e., the reduction in the number of consumers who buy new durable goods $\Delta q_{sn}^{*}$ is half of the total reduction in the number of consumers $\Delta q_{s}^{*}$. When the carbon trading price $p_{e}=3.97$, $\theta_{s1}^{*}=\theta_{s2}^{*}$, which means the utility value of consumers who buy new durable goods is equal to the utility value of consumers who buy used durable goods. At this point, the consumers in the market all will choose to buy new durable goods.

**Figure 5:**
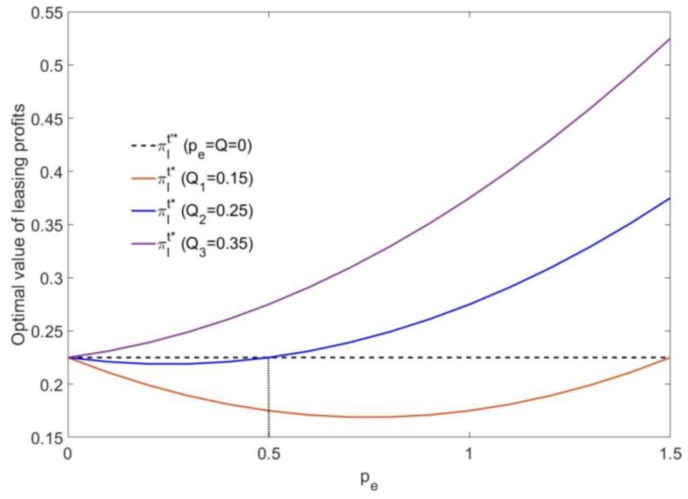
The effect of $p_{e}$ on the optimal leasing profit.

Figure 5 shows the following: If the mixed carbon policy is not implemented, the manufacturer’s leasing profit is a fixed value $\pi_{l}^{\prime *}=0.225$. If the mixed carbon policy is implemented, both the carbon emissions cap *Q* and the carbon trading price $p_{e}$ affect the manufacturer’s leasing profit. The larger the carbon emission cap *Q* is, the higher the manufacturer’s leasing profit. As the $p_{e}$ increases, the manufacturer’s leasing profit will decrease at first and then increase, but it will always be lower than 0.225 when *Q* = 0.15. In addition, when *Q* = 0.25, note the following: the leasing profit $\pi_{l}^{*}<\pi_{l}^{\prime *}=0.225$ when $0<p_{e}<0.5$. When $p_{e}>0.5$, the leasing profit will increase continuously as the carbon trading price $p_{e}$ increases. The leasing profit is always higher than 0.225 when *Q* = 0.35, and as the carbon trading price $p_{e}$ increases, the manufacturer’s leasing profit increases at an exponential rate.

**Figure 6:**
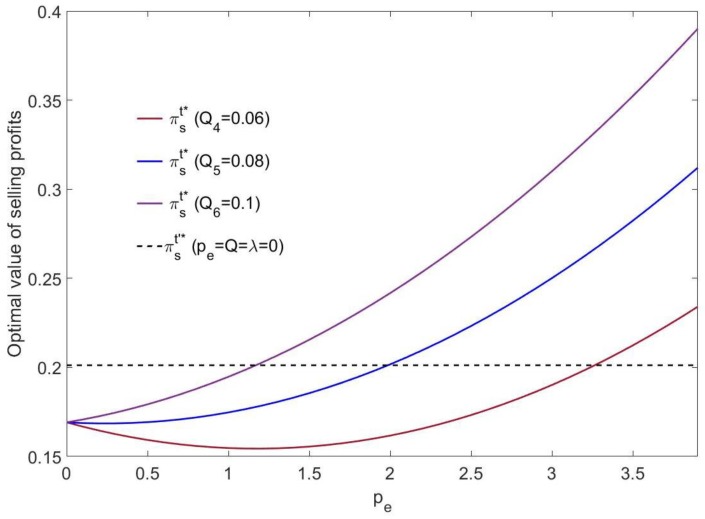
The effect of $p_{e}$ on the optimal selling profit ($\lambda_{2}=0.2$).

As shown in Figure 6, if the mixed carbon policy is not implemented, the manufacturer’s selling profit is a fixed value $\pi_{s}^{\prime *}=0.2$. If the mixed carbon policy is implemented, the higher the carbon cap value is, the faster the manufacturer’s selling profit growth rate. The higher the carbon cap value is, the earlier the selling profit exceeds the fixed value $\pi_{s}^{\prime *}=0.2$. Regardless of the value of the carbon emissions cap, with the increase of carbon trading price $p_{e}$, the selling profit will be higher than 0.2 eventually. When the carbon cap value is low (*Q* = 0.06), the manufacturer’s selling profit will decrease at first and then increase with the increase of carbon trading price $p_{e}$. When the carbon cap value is high (*Q* = 0.1), the selling profit will increase continuously as the carbon trading price $p_{e}$ increases.

**Figure 7:**
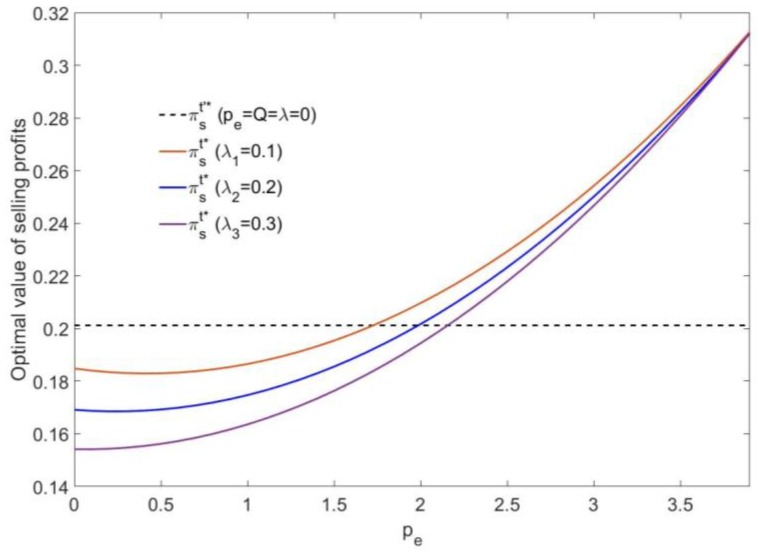
The effect of $p_{e}$ on the optimal selling profit (*Q*_5_ = 0.08).

According to Figure 7, if the mixed carbon policy is not implemented, the manufacturer’s selling profit is a fixed value $\pi_{s}^{\prime *}=0.2$. If the mixed carbon policy is implemented, the increase of the carbon trading price $p_{e}$ will lead to an increase in the selling profit of the manufacturer. In addition, the higher the carbon tax rate λ is, the lower the initial selling profit, but with the increase of the carbon trading price $p_{e}$, the growth in the profit is faster than the growth in the profit under a low carbon tax rate.

Moreover, the selling profit is lower than $\pi_{s}^{\prime *}=0.2$ when the carbon trading price is low. Only when the carbon trading price is high the manufacturer can gain more profit than the profit that would have been gained if the mixed carbon policy was not implemented. In addition, the selling profit is growing closer to $p_{e}Q$ as the carbon trading price increases.

**Figure 8:**
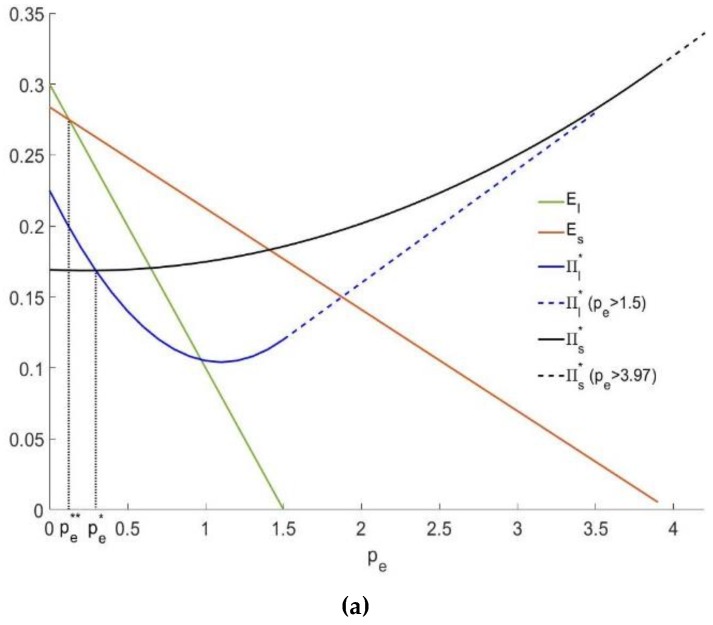

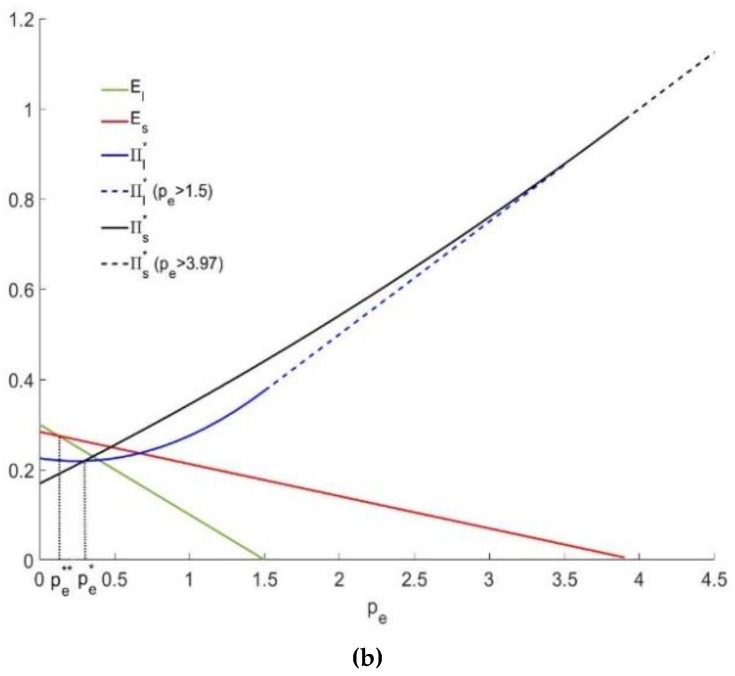
The effect of $p_{e}$ on the optimal profit and total carbon emissions. (**a**) when *Q*_5_ = 0.08; (**b**) when *Q*_2_ = 0.25.

In Figure 8a,b, whether the carbon emission cap is high or low, we can obtain the following facts: as the carbon trading price increases, the total carbon emissions under leasing and selling will both be reduced, and the carbon emissions under leasing will be reduced faster. When the carbon trading price $p_{e}=p_{e}^{**}$, the total carbon emissions under leasing and selling are the same $E_{l}=E_{s}$. When the carbon trading price is low, the profit may decrease at first, but as the carbon trading price increases, the profit under leasing and selling will both eventually increase. The profit under leasing and selling will be equal to $p_{e}Q$ when the carbon trading price is large enough. When the carbon trading price $p_{e}=p_{e}^{*}$, the profit under leasing and selling is the same $\pi_{l}^{*}=\pi_{s}^{*}$. Therefore, when $p_{e}^{**}<p_{e}<p_{e}^{*}$, the leasing profit is higher than the selling profit $\pi_{l}^{*}>\pi_{s}^{*}$, and at the same time, the total carbon emissions under leasing are lower than those under selling $E_{l}<E_{s}$. Thus, we can achieve a win-win result by controlling the carbon trading price within the interval $\left[p_{e}^{**},p_{e}^{*}\right]$.

## 7. Conclusions

With the promotion of various carbon policies in many nations, it is imperative for the durable goods manufacturers with high carbon emissions to adjust their marketing decisions. Much attention has been paid to carbon emissions in the production process. However, many durable goods actually produce higher carbon emissions in the consumption process. Previous studies have focused on the production, pricing and emissions reduction of manufacturers under the constraint of carbon emissions in the production process. Therefore, in light of the idea that carbon emissions in both production and consumption should be constrained by carbon policies, this paper describes a situation in which a monopoly manufacturer produces and offers (leases or sells) a single durable goods to consumers and in which the durable goods produce high carbon emissions in both the production and consumption process. The aim of this paper is to find the best marketing model for manufacturers and the optimal ranges for the carbon trading price, the carbon cap and the carbon tax rate for the government. Some interesting managerial implications obtained from the propositions and the numerical analysis are as follows:

(1) Production strategies: The mixed carbon policy dampens the manufacturer’s incentive to produce, and when the carbon trading price is too high, the manufacturer does not carry out production activities. Whether under leasing or selling situation, the manufacturer will only carry on the production activity when the carbon trading price is lower than the threshold value.
(2) Consumption behaviour: The mixed carbon policy restrains the consumers’ consuming behaviour. The number of consumers participating in the market both decreases, and the decreasing rate is the same. Moreover, in regulating consumer behaviours, the carbon tax is more effective than the carbon price.
(3) Marketing model selections: The carbon cap *Q* and the carbon tax rate *λ* cannot affect the manufacturer’s selection of a leasing and a selling model. When the carbon trading price is lower than the threshold, leasing is the optimal marketing model; when the carbon trading price is higher than the threshold, selling is the optimal marketing model.
(4) The carbon cap setting: A carbon cap *Q* that is too low will severely decrease the manufacturer’s incentive to produce, but a carbon cap *Q* that is too high will not achieve the government’s goal of controlling carbon emissions. The manufacturer can be encouraged to reduce the carbon emissions and meanwhile, its enthusiasm for production will not be overly affected only when the carbon cap *Q* is on a specific interval.
(5) Win-win results: The leasing model can create a win-win result between the manufacturer’s profits and a friendly environment under certain conditions: when the carbon trading price in the carbon market is lower than, the government should keep the carbon tax rate below the threshold value $\frac{\left[\left(1+3\delta \right)\left(1+\rho \delta \right)-\left(1+\delta +2\rho \delta \right)\left(1+\delta \right)\right]v}{\left(1+3\delta \right)(e_{1}+\rho e_{2})}$; as the carbon trading price rises, when $p_{e}^{**}<p_{e}<p_{e}^{*}$, the government should keep the carbon tax rate higher than the threshold value $\frac{\left[\left(1+3\delta \right)\left(1+\rho \delta \right)-\left(1+\delta +2\rho \delta \right)\left(1+\delta \right)\right]v}{\left(1+3\delta \right)(e_{1}+\rho e_{2})}$.

There are some limitations of our study that should be considered in future research. First, our study examines the impact of the mixed carbon policy on a durable goods manufacturer in a monopolistic market. Further investigation could study how the mixed carbon policy affects the durable goods manufacturer’s decision in a competitive market. Second, we do not consider the green technology investment of the manufacturer under the constraint of the mixed carbon policy. It could be more interesting if the green technology investment is considered in our model.

## Figures and Tables

**Table 1:** The major notations and definition.

Notation and Parameters	Definition
*n*	New product
*u*	Used product
*ν*	Quality of the new durable goods
*δ*	Product durability
*l*	Leasing strategy
*s*	Selling strategy
*p_e_*	Unit carbon trading price
*Q*	Carbon emissions cap
*λ*	Carbon tax of per unit carbon emissions
*θ*	Consumers’ willingness to pay (consumer type)
*ρ*	Discount factor of revenues or cash flows
*t*	Model periods
*e_i_*, *i* ∈ (0,1,2)	Carbon emissions of unit product in production and in two consumption processes, respectively.
*p_li_*, *i* ∈(*n*,*u*)	Leasing prices of new products and used products
*p_si_*, *i* ∈ (*n*,*u*)	Selling prices of new products and used products
*u_li_*(*θ*), *i* ∈ (*n*,*u*,0)	Net utility for the different consumer type *θ* under leasing
*u_si_*(*θ*), *i* ∈ (*n*,*u*,0)	Net utility for the different consumer type θ under selling
$E_{i}^{*}$, *i* ∈ (*l*,*s*)	Actual total carbon emissions of leasing and selling with the mixed carbon policy
$E_{i}^{\prime }$, *i* ∈ (*l*,*s*)	Actual total carbon emissions of leasing and selling without the mixed carbon policy
Decision variables	
$q_{\ln}^{*}$, $q_{sn}^{*}$	Manufacturing quantity under leasing and selling with the mixed carbon policy
$q_{\ln}^{\prime }$, $q_{sn}^{\prime }$	Manufacturing quantity under leasing and selling without the mixed carbon policy
Objective function	
$\pi_{i}^{*}$, *i* ∈ (*l*,*s*)	Leasing profit and selling profit of the manufacturer with the mixed carbon policy
$\pi_{i}^{\prime }$, *i* ∈ (*l*,*s*s)	Leasing profit and selling profit of the manufacturer without the mixed carbon policy

## Appendix A. Proof of Proposition 1

According to the necessary conditions for the Equations (8) and (18), we can obviously obtain that: when $p_{e}>\max\{\frac{\left(1+\delta \right)v}{e_{0}+e_{1}+e_{2}},\frac{\left(1+\rho \delta \right)v-\lambda \left(e_{1}+\rho e_{2}\right)}{e_{0}}\}$, we have $q_{\ln}^{t*}=q_{sn}^{t*}=0$;

When $p_{e}<\min\{\frac{\left(1+\delta \right)v}{e_{0}+e_{1}+e_{2}},\frac{\left(1+\rho \delta \right)v-\lambda \left(e_{1}+\rho e_{2}\right)}{e_{0}}\}$, we have $q_{sn}^{t*}>0$, $q_{ln}^{t*}>0$.

By comparing $\frac{\left(1+\delta \right)v}{e_{0}+e_{1}+e_{2}}$ and $\frac{\left(1+\rho \delta \right)v-\lambda \left(e_{1}+\rho e_{2}\right)}{e_{0}}$, we obtain the following:

(1) If the carbon tax rate $\lambda <\frac{\left(1+\rho \delta \right)\left(e_{1}+e_{2}\right)-\left(1-\rho \right)\delta e_{0}}{\left(e_{1}+\rho e_{2}\right)\left(e_{0}+e_{1}+e_{2}\right)}v$, we have $\frac{\left(1+\delta \right)v}{e_{0}+e_{1}+e_{2}}<\frac{\left(1+\rho \delta \right)v-\lambda \left(e_{1}+\rho e_{2}\right)}{e_{0}}$. Then when $\frac{\left(1+\delta \right)v}{e_{0}+e_{1}+e_{2}}<p_{e}<\frac{\left(1+\rho \delta \right)v-\lambda \left(e_{1}+\rho e_{2}\right)}{e_{0}}$, we have $q_{sn}^{t*}>q_{ln}^{t*}=0$.
(2) If the carbon tax rate $\lambda >\frac{\left(1+\rho \delta \right)\left(e_{1}+e_{2}\right)-\left(1-\rho \right)\delta e_{0}}{\left(e_{1}+\rho e_{2}\right)\left(e_{0}+e_{1}+e_{2}\right)}v$, We have $\frac{\left(1+\rho \delta \right)v-\lambda \left(e_{1}+\rho e_{2}\right)}{e_{0}}<\frac{\left(1+\delta \right)v}{e_{0}+e_{1}+e_{2}}$. Then when $\frac{\left(1+\rho \delta \right)v-\lambda \left(e_{1}+\rho e_{2}\right)}{e_{0}}<p_{e}<\frac{\left(1+\delta \right)v}{e_{0}+e_{1}+e_{2}}$, we have $q_{ln}^{t*}>q_{sn}^{t*}=0$.

Then Proposition 1 is proved.

## Appendix B. Proof of Proposition 2

By comparing the reduction quantity of the new products $\Delta q_{\ln}^{*}$, the used products $\Delta q_{lu}^{*}$ and the total products $\Delta q_{l}^{*}$ under the mixed carbon policy, we can readily observe that:

$$\Delta q_{l}^{*}=\frac{p_{e}\left(e_{0}+e_{1}+e_{2}\right)}{\left(1+3\delta \right)v}=2\Delta q_{\ln}^{*}=2\Delta q_{lu}^{*}>0$$

Taking the first-order derivatives of $\Delta q_{\ln}^{*}$ and $\Delta q_{lu}^{*}$ with respect to $p_{e}$, and we have:

$$\frac{\partial \Delta q_{\ln}^{*}}{\partial p_{e}}=\frac{\partial \Delta q_{lu}^{*}}{\partial p_{e}}=\frac{e_{0}+e_{1}+e_{2}}{2\left(1+3\delta \right)v}>0$$

Then Proposition 2 is proved.

## Appendix C. Proof of Proposition 3

By comparing the reduction quantity of the new products $\Delta q_{sn}^{*}$, the used products $\Delta q_{su}^{*}$ and the total products $\Delta q_{s}^{*}$, we can readily observe that:

$$\Delta q_{s}^{*}=\frac{p_{e}e_{0}+\lambda \left(e_{1}+\rho e_{2}\right)}{\left(1+\delta +2\rho \delta \right)v}=2\Delta q_{sn}^{*}=2\Delta q_{su}^{*}>0$$

Taking the first-order derivatives of $\Delta q_{sn}^{*}$ and $\Delta q_{su}^{*}$ with respect to $p_{e}$ and *λ*, and we have:

$$\frac{\partial \Delta q_{sn}^{*}}{\partial p_{e}}=\frac{\partial \Delta q_{su}^{*}}{\partial p_{e}}=\frac{e_{0}}{2\left(1+\delta +2\rho \delta \right)v}>0$$

$$\frac{\partial \Delta q_{sn}^{*}}{\partial \lambda }=\frac{\partial \Delta q_{su}^{*}}{\partial \lambda }=\frac{e_{1}+\rho e_{2}}{2\left(1+\delta +2\rho \delta \right)v}>0$$

According to the assumption $e_{2}\geq e_{1}\geq e_{0}$, we can obtain the following:

$$\frac{\partial \Delta q_{sn}^{*}}{\partial \lambda }=\frac{\partial \Delta q_{su}^{*}}{\partial \lambda }>\frac{\partial \Delta q_{sn}^{*}}{\partial p_{e}}=\frac{\partial \Delta q_{su}^{*}}{\partial p_{e}}$$

Then Proposition 3 is proved.

## Appendix D. Proof of Proposition 4

Substituting Equation (8) into Equations (30) and (31), we can obtain the following:

$$\begin{array}{ll} \Delta \pi_{l} & =\pi_{l}^{\prime *}-\pi_{l}^{t*}=\frac{2(1+\delta )v-p_{e}\left(e_{0}+e_{1}+e_{2}\right)}{4\left(1+3\delta \right)v}p_{e}\left(e_{0}+e_{1}+e_{2}\right)-p_{e}Q\\ & =p_{e}\left[\left(e_{0}+e_{1}+e_{2}\right)q_{\ln}^{t*}+\frac{p_{e}{\left(e_{0}+e_{1}+e_{2}\right)}^{2}}{4\left(1+3\delta \right)v}-Q\right]=p_{e}\left(Q_{A}-Q\right) \end{array}$$

let $Q_{A}=\left(e_{0}+e_{1}+e_{2}\right)q_{\ln}^{t*}+\frac{p_{e}{\left(e_{0}+e_{1}+e_{2}\right)}^{2}}{4\left(1+3\delta \right)v}$.

Taking the first-order derivatives of $\Delta \pi_{l}$ with respect to $p_{e}$, and we have:

$$\begin{array}{ll} \frac{\partial \Delta \pi_{l}}{\partial p_{e}} & =\frac{(1+\delta )v-p_{e}\left(e_{0}+e_{1}+e_{2}\right)}{2\left(1+3\delta \right)v}\left(e_{0}+e_{1}+e_{2}\right)-Q\\ & =\left(e_{0}+e_{1}+e_{2}\right)q_{\ln}^{t*}-Q \end{array}$$

We can derive the following results obviously:

When $Q<\left(e_{0}+e_{1}+e_{2}\right)q_{\ln}^{t*}$, we have $\Delta \pi_{l}>0$, $\frac{\partial \Delta \pi_{l}}{\partial p_{e}}>0$; when $\left(e_{0}+e_{1}+e_{2}\right)q_{\ln}^{t*}<Q<Q_{A}$, we have $\Delta \pi_{l}>0$, $\frac{\partial \Delta \pi_{l}}{\partial p_{e}}<0$; when $Q>Q_{A}$, we have $\Delta \pi_{l}<0$, $\frac{\partial \Delta \pi_{l}}{\partial p_{e}}<0$.

Then Proposition 4 is proved.

## Appendix E. Proof of Proposition 5

Substituting Equation (18) into Equations (32–34), we can obtain the following:

$$\begin{array}{ll} \Delta \pi_{s}=\pi_{s}^{\prime *}-\pi_{s}^{t*} & =\frac{2\left(1+\rho \delta \right)v-p_{e}e_{0}-\lambda \left(e_{1}+\rho e_{2}\right)}{4\left(1+\delta +2\rho \delta \right)v}\left(p_{e}e_{0}+\lambda e_{1}+\rho \lambda e_{2}\right)-p_{e}Q\\ & =\left(p_{e}e_{0}+\lambda e_{1}+\rho \lambda e_{2}\right)q_{sn}^{t*}+\frac{{\left(p_{e}e_{0}+\lambda e_{1}+\rho \lambda e_{2}\right)}^{2}}{4\left(1+\delta +2\rho \delta \right)v}-p_{e}Q=p_{e}\left(Q_{B}-Q\right) \end{array}$$

let $Q_{B}=\frac{\left(p_{e}e_{0}+\lambda e_{1}+\rho \lambda e_{2}\right)q_{sn}^{t*}}{p_{e}}+\frac{{\left(p_{e}e_{0}+\lambda e_{1}+\rho \lambda e_{2}\right)}^{2}}{4p_{e}\left(1+\delta +2\rho \delta \right)v}$.

Taking the first-order derivatives of $\Delta \pi_{s}$ with respect to $p_{e}$ and *λ*, and we have:

$$\begin{array}{ll} \frac{\partial \Delta \pi_{s}}{\partial p_{e}} & =\frac{\left(1+\rho \delta \right)v-p_{e}e_{0}-\lambda \left(e_{1}+\rho e_{2}\right)}{2\left(1+\delta +2\rho \delta \right)v}e_{0}-Q\\ & =e_{0}q_{sn}^{t*}-Q \end{array}$$

$$\begin{array}{ll} \frac{\partial \Delta \pi_{s}}{\partial \lambda } & =\frac{\left(1+\rho \delta \right)v-p_{e}e_{0}-\lambda \left(e_{1}+\rho e_{2}\right)}{2\left(1+\delta +2\rho \delta \right)v}\left(e_{1}+\rho e_{2}\right)\\ & =\left(e_{1}+\rho e_{2}\right)q_{sn}^{t*}>0 \end{array}$$

We can derive the following results obviously:

When $Q<e_{0}q_{sn}^{t*}$, we have $\Delta \pi_{s}>0$, $\frac{\partial \Delta \pi_{s}}{\partial p_{e}}>0$; when $e_{0}q_{sn}^{t*}<Q<Q_{B}$, we have $\Delta \pi_{s}>0$, $\frac{\partial \Delta \pi_{s}}{\partial p_{e}}<0$; when $Q>Q_{B}$, we have $\Delta \pi_{s}<0$, $\frac{\partial \Delta \pi_{s}}{\partial p_{e}}<0$.

Then Proposition 5 is proved.

## Appendix F. Proof of Proposition 6

By comparing Equations (11) and (21), we can obtain the following:

$$\pi_{l}^{t*}-\pi_{s}^{t*}=\frac{{\left[(1+\delta )v-p_{e}\left(e_{0}+e_{1}+e_{2}\right)\right]}^{2}}{4\left(1+3\delta \right)v}-\frac{{\left[\left(1+\rho \delta \right)v-p_{e}e_{0}-\lambda \left(e_{1}+\rho e_{2}\right)\right]}^{2}}{4\left(1+\delta +2\rho \delta \right)v}$$

Due to $\pi_{l}^{t*}>0$, $\pi_{s}^{t*}>0$, to obtain the results easily, we compare the square root of the profits of the manufacturer under leasing and selling, and we can compute the difference as the following:

$$\begin{array}{ll} \sqrt{\pi_{l}^{t*}}-\sqrt{\pi_{s}^{t*}} & =\frac{(1+\delta )v-p_{e}\left(e_{0}+e_{1}+e_{2}\right)}{2\sqrt{\left(1+3\delta \right)v}}-\frac{\left(1+\rho \delta \right)v-p_{e}e_{0}-\lambda \left(e_{1}+\rho e_{2}\right)}{2\sqrt{\left(1+\delta +2\rho \delta \right)v}}\\ & =\frac{\left[(1+\delta )\sqrt{\left(1+\delta +2\rho \delta \right)}-\left(1+\rho \delta \right)\sqrt{\left(1+3\delta \right)}\right]v}{2\sqrt{\left(1+3\delta \right)\left(1+\delta +2\rho \delta \right)v}}+\\ & \;\frac{\sqrt{1+3\delta }(e_{1}+\rho e_{2})\lambda -\left[\sqrt{1+\delta +2\rho \delta }(e_{0}+e_{1}+e_{2})-\sqrt{1+3\delta }e_{0}\right]p_{e}}{2\sqrt{\left(1+3\delta \right)\left(1+\delta +2\rho \delta \right)v}} \end{array}$$

Due to $(1+\delta )\sqrt{\left(1+\delta +2\rho \delta \right)}>0$, $\left(1+\rho \delta \right)\sqrt{\left(1+3\delta \right)}>0$, we compare the square of the $(1+\delta )\sqrt{\left(1+\delta +2\rho \delta \right)}$ and $\left(1+\rho \delta \right)\sqrt{\left(1+3\delta \right)}$, and we have:

$$\begin{array}{l} {\left(1+\delta \right)}^{2}\left(1+\delta +2\rho \delta \right)-{\left(1+\rho \delta \right)}^{2}\left(1+3\delta \right)\\ =\delta \left[(1-\rho^{2}\delta )\left(1+3\delta \right)-(1-\delta )\left(1+\delta +2\rho \delta \right)\right] \end{array}$$

According to the assumption 0<ρ<1, we have $\left(1-\rho^{2}\delta )>(1-\delta \right)$, (1+3δ)>(1+δ+2ρδ), thus $(1-\rho^{2}\delta )\left(1+3\delta \right)-(1-\delta )\left(1+\delta +2\rho \delta \right)>0$. Thereby, we calculate that $(1+\delta )\sqrt{\left(1+\delta +2\rho \delta \right)}-\left(1+\rho \delta \right)\sqrt{\left(1+3\delta \right)}>0$. In addition, according to the assumption $e_{2}\geq e_{1}\geq e_{0}$, we have $\sqrt{1+\delta +2\rho \delta }(e_{0}+e_{1}+e_{2})-\sqrt{1+3\delta }e_{0}>0$. Then we can obtain the following results:

When the carbon trading price $0<p_{e}<p_{e}^{*}$, we have $\sqrt{\pi_{l}^{t*}}-\sqrt{\pi_{s}^{t*}}>0$, thereby $\pi_{l}^{t*}>\pi_{s}^{t*}$. When the carbon trading price $p_{e}>p_{e}^{*}$, we have $\sqrt{\pi_{l}^{t*}}-\sqrt{\pi_{s}^{t*}}<0$, thereby $\pi_{l}^{t*}<\pi_{s}^{t*}$.

Let $p_{e}^{*}=\frac{\left[(1+\delta )\sqrt{1+\delta +2\rho \delta }-(1+\rho \delta )\sqrt{1+3\delta }\right]v+\sqrt{1+3\delta }(e_{1}+\rho e_{2})\lambda }{\sqrt{1+\delta +2\rho \delta }(e_{0}+e_{1}+e_{2})-\sqrt{1+3\delta }e_{0}}$.

Then Proposition 6 is proved.

## Appendix G. Proof of Proposition 7

Comparing the actual total carbon emissions of leasing and selling, according to Equations (8) and (18), we have:

$$\begin{array}{ll} E_{l}-E_{s} & =\left(e_{0}+e_{1}+e_{2}\right)(q_{\ln}^{t*}-q_{sn}^{t*})\\ & =\left(e_{0}+e_{1}+e_{2}\right)\frac{-\left[\left(1+3\delta \right)\left(1+\rho \delta \right)-\left(1+\delta +2\rho \delta \right)\left(1+\delta \right)\right]v}{2\left(1+3\delta \right)\left(1+\delta +2\rho \delta \right)v}+\\ & \;\left(e_{0}+e_{1}+e_{2}\right)\frac{\left(1+3\delta \right)(e_{1}+\rho e_{2})\lambda -\left[\left(1+\delta +2\rho \delta \right)(e_{0}+e_{1}+e_{2})-\left(1+3\delta \right)e_{0}\right]p_{e}}{2\left(1+3\delta \right)\left(1+\delta +2\rho \delta \right)v} \end{array}$$

Simplify (1+3δ)(1+ρδ)−(1+δ+2ρδ)(1+δ), according to the assumption 0<ρ<1, 0<δ<1, we have:

(1+3δ)(1+ρδ)−(1+δ+2ρδ)(1+δ)=δ(1−ρ)(1−δ)>0

and according to the assumption $e_{2}\geq e_{1}\geq e_{0}$, we have $\left(1+\delta +2\rho \delta \right)(e_{0}+e_{1}+e_{2})-\left(1+3\delta \right)e_{0}>0$.

Then we can obtain the following results:

When the carbon tax rate $\lambda <\frac{\left[\left(1+3\delta \right)\left(1+\rho \delta \right)-\left(1+\delta +2\rho \delta \right)\left(1+\delta \right)\right]v}{\left(1+3\delta \right)(e_{1}+\rho e_{2})}$, then $E_{l}-E_{s}<0$.

Then Proposition 7 (1) is proved.

If the carbon tax rate $\lambda >\frac{\left[\left(1+3\delta \right)\left(1+\rho \delta \right)-\left(1+\delta +2\rho \delta \right)\left(1+\delta \right)\right]v}{\left(1+3\delta \right)(e_{1}+\rho e_{2})}$: when $p_{e}<p_{e}^{**}$, we have $E_{l}-E_{s}>0$; when $p_{e}=p_{e}^{**}$, we have $E_{l}-E_{s}=0$; when $p_{e}>p_{e}^{**}$, we have $E_{l}-E_{s}<0$.

Then Proposition 7 (2) is proved.

Let $p_{e}^{**}=\frac{\left(1+3\delta \right)(e_{1}+\rho e_{2})\lambda -\left[\left(1+3\delta \right)\left(1+\rho \delta \right)-\left(1+\delta +2\rho \delta \right)\left(1+\delta \right)\right]v}{\left(1+\delta +2\rho \delta \right)(e_{0}+e_{1}+e_{2})-\left(1+3\delta \right)e_{0}}$.

## Appendix H. Proof of Proposition 8

Combining the results in Appendix F and Appendix G, we can obtain the following:

If the carbon tax rate $\lambda <\frac{\left[\left(1+3\delta \right)\left(1+\rho \delta \right)-\left(1+\delta +2\rho \delta \right)\left(1+\delta \right)\right]v}{\left(1+3\delta \right)(e_{1}+\rho e_{2})}$: when $0<p_{e}<p_{e}^{*}$, we have $\pi_{l}^{t*}>\pi_{s}^{t*}$ and $E_{l}<E_{s}$; when $p_{e}>p_{e}^{*}$, we have $\pi_{l}^{t*}<\pi_{s}^{t*}$ and $E_{l}<E$.

Then Proposition 8 (1) is proved.

Comparing the value of $p_{e}^{*}$ and $p_{e}^{**}$, and we have:

$$\begin{array}{l} p_{e}^{*}-p_{e}^{**}\\ =\frac{\left[(1+\delta )\sqrt{1+\delta +2\rho \delta }-(1+\rho \delta )\sqrt{1+3\delta }\right]v+\sqrt{1+3\delta }(e_{1}+\rho e_{2})\lambda }{\sqrt{1+\delta +2\rho \delta }(e_{0}+e_{1}+e_{2})-\sqrt{1+3\delta }e_{0}}-\\ \frac{\left(1+3\delta \right)(e_{1}+\rho e_{2})\lambda -\left[\left(1+3\delta \right)\left(1+\rho \delta \right)-\left(1+\delta +2\rho \delta \right)\left(1+\delta \right)\right]v}{\left(1+\delta +2\rho \delta \right)(e_{0}+e_{1}+e_{2})-\left(1+3\delta \right)e_{0}}\\ =\frac{\left[(1+\delta )a-(1+\rho \delta )b\right]v+b(e_{1}+\rho e_{2})\lambda }{a(e_{0}+e_{1}+e_{2})-be_{0}}-\frac{\left[a^{2}\left(1+\delta \right)-\left(1+\rho \delta \right)b^{2}\right]v+b^{2}(e_{1}+\rho e_{2})\lambda }{a^{2}(e_{0}+e_{1}+e_{2})-b^{2}e_{0}} \end{array}$$

let $a=\sqrt{1+\delta +2\rho \delta }$, $b=\sqrt{1+3\delta }$. According to the assumption 0<ρ<1, 0<δ<1, we have 1<a<b<2, (1+δ)a−(1+ρδ)b>0, $a^{2}\left(1+\delta \right)-\left(1+\rho \delta \right)b^{2}<0$. we can determine that $p_{e}^{*}-p_{e}^{**}>0$.

Then if the carbon tax rate $\lambda >\frac{\left[\left(1+3\delta \right)\left(1+\rho \delta \right)-\left(1+\delta +2\rho \delta \right)\left(1+\delta \right)\right]v}{\left(1+3\delta \right)(e_{1}+\rho e_{2})}$: when $p_{e}<p_{e}^{**}$, we have $\pi_{l}^{t*}>\pi_{s}^{t*}$ and $E_{l}>E_{s}$; when $p_{e}^{**}<p_{e}<p_{e}^{*}$, we have $\pi_{l}^{t*}>\pi_{s}^{t*}$ and $E_{l}<E_{s}$; when $p_{e}>p_{e}^{*}$, we have $\pi_{l}^{t*}<\pi_{s}^{t*}$ and $E_{l}<E_{s}$.

Then Proposition 8 (2) is proved.
